# Redox-Controlled Shunts in a Synthetic Chemical Reaction Cycle

**DOI:** 10.1021/jacs.3c00985

**Published:** 2023-04-24

**Authors:** Anastasiia Sharko, Benjamin Spitzbarth, Thomas M. Hermans, Rienk Eelkema

**Affiliations:** † University of Strasbourg & CNRS, UMR7140, 67083 Strasbourg, France; ‡ Department of Chemical Engineering, Delft University of Technology, Van der Maasweg 9, 2629 HZ Delft, The Netherlands

## Abstract

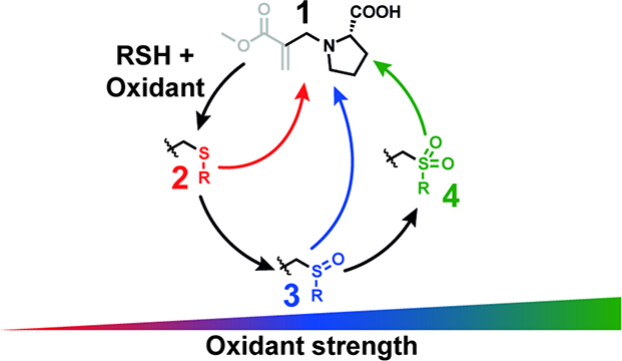

Shunts, alternative pathways in chemical reaction networks (CRNs), are ubiquitous in nature, enabling adaptability to external and internal stimuli. We introduce a CRN in which the recovery of Michael-accepting species is driven by oxidation chemistry. Using weak oxidants can enable access to two shunts within this CRN with different kinetics and a reduced number of side reactions compared to the main cycle that is driven by strong oxidants. Furthermore, we introduce a strategy to recycle one of the main products under flow conditions to partially reverse the CRN and control product speciation throughout time. These findings introduce new levels of control over artificial CRNs, driven by redox chemistry, narrowing the gap between synthetic and natural systems.

## Introduction

Nature has evolved myriad ways of performing chemical conversions to regulate living organisms. Such conversions often are done in chemical reaction networks (CRNs), where intricate connections between reactants and products exist. One of the key reaction cycles to control cellular respiration is the Krebs cycle, in which high-energy molecules (NADH, GTP, and QH_2_) are generated by transforming acetyl-CoA into carbon dioxide in eight consecutive reactions;^1^ however, under oxidative stress or carbon feedstock shortage, three of them are bypassed. This alternative pathway that allows respiration to continue is called the glyoxylate shunt.^2^ Similar shunts, such as the P450 peroxide shunt,^3^ GABA shunt,^4^ and pentose phosphate shunt,^5^ among others,^1,6^ are ubiquitous in metabolism and are responsible for their adaptive regulation. A distinct feature of natural shunts is that they offer control over product speciation in response to external stimuli or the organism’s internal needs.^7−10^ Although there are numerous artificial reaction networks of various complexity,^11−20^ shunts have not yet been explored in the context of systems chemistry.

**Figure 1:**
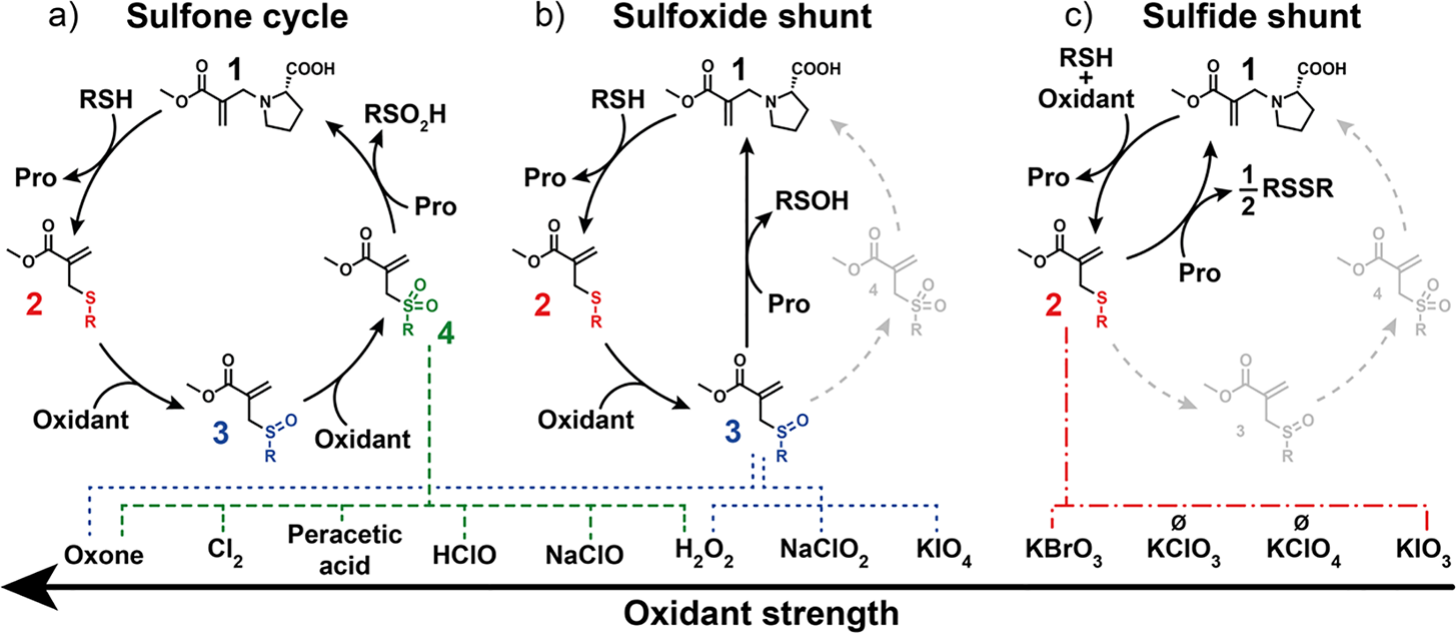
Simplified CRN schemes; depending on the oxidant strength, proline-MA **1** recovery goes via the sulfone cycle (a) with strong oxidants and two different shunts (b,c) with weaker oxidants, leading to different sulfur product speciations. Here, Pro is l-proline, and R = 4-carboxyphenyl.

Here, we show how two alternative pathways in an artificial reaction cycle (i.e., shunts) can be accessed depending on the oxidant strength (Figure 1). For the sake of clarity in the following discussion, we define shunt as an alternative pathway in a CRN with distinct intermediate species that becomes available or even dominant upon changing external conditions (e.g., reactant concentration, catalytic activity, oxidant strength, and pH, but excluding temperature or pressure). In our current reaction cycle, we start from a Michael acceptor (MA) that reacts with a thiol to release proline. The thiol adduct is subsequently oxidized to the sulfoxide and further to the sulfone, allowing the proline to re-attack and recover the original MA species. Strong oxidants provide access to all oxidation states of the sulfur species, i.e., sulfide, sulfoxide, and sulfone, defining the maximum speciation of the network (Figure 1a). For weaker oxidants, the sulfone pathway becomes less dominant, which we refer to as the “sulfoxide shunt” (Figure 1b). For the weakest oxidants, only the sulfide is available (i.e., the “sulfide shunt”, Figure 1c), which leads to the smallest CRN with the fewest side reactions and the slowest kinetics (Table S2). Finally, we show how a reductant can partially reverse the sulfoxide shunt by recycling the disulfide product, resembling the partial reversibility^21−23^ of the Krebs cycle in the presence of primordial reducing agents such as cyanide or hydrogen.

## Results and Discussion

### Choice of Chemistry and Reagents

Conjugate additions to MAs have been employed for efficient functionalization reactions in a vast range of applications, such as in material science and biofunctionalizations.^24−31^ In their previous work, the Eelkema group attempted to design a redox-controlled reaction cycle for the recovery of MAs based on thiol-addition and -elimination chemistry. Although the steps of this cycle worked well in isolation, combining them led to challenges such as over-oxidation and significant side reactivity of the waste products.^32^ We decided to work with different oxidants to overcome the challenges faced previously and to look at a different class of MAs. β’-Substituted MAs are especially attractive due to their ability to retain the reactive double bond upon the addition of a nucleophile and subsequent elimination of a leaving group in the β’-position.^33−38^ The group of Thayumanavan demonstrated the addition of thiols on amine-functionalized β’-MAs for application in chemical switches.^39^ Furthermore, it was found that the more oxidized sulfone adducts are electron-deficient enough to react with amines to form amine-functionalized MAs.^34,35,40^ Therefore, we hypothesized that oxidation of the thiol-functionalized MA would enable the completion of a reaction cycle, where the initial MA is recovered (Figure 1).

### Sulfone Cycle

**Figure 2:**
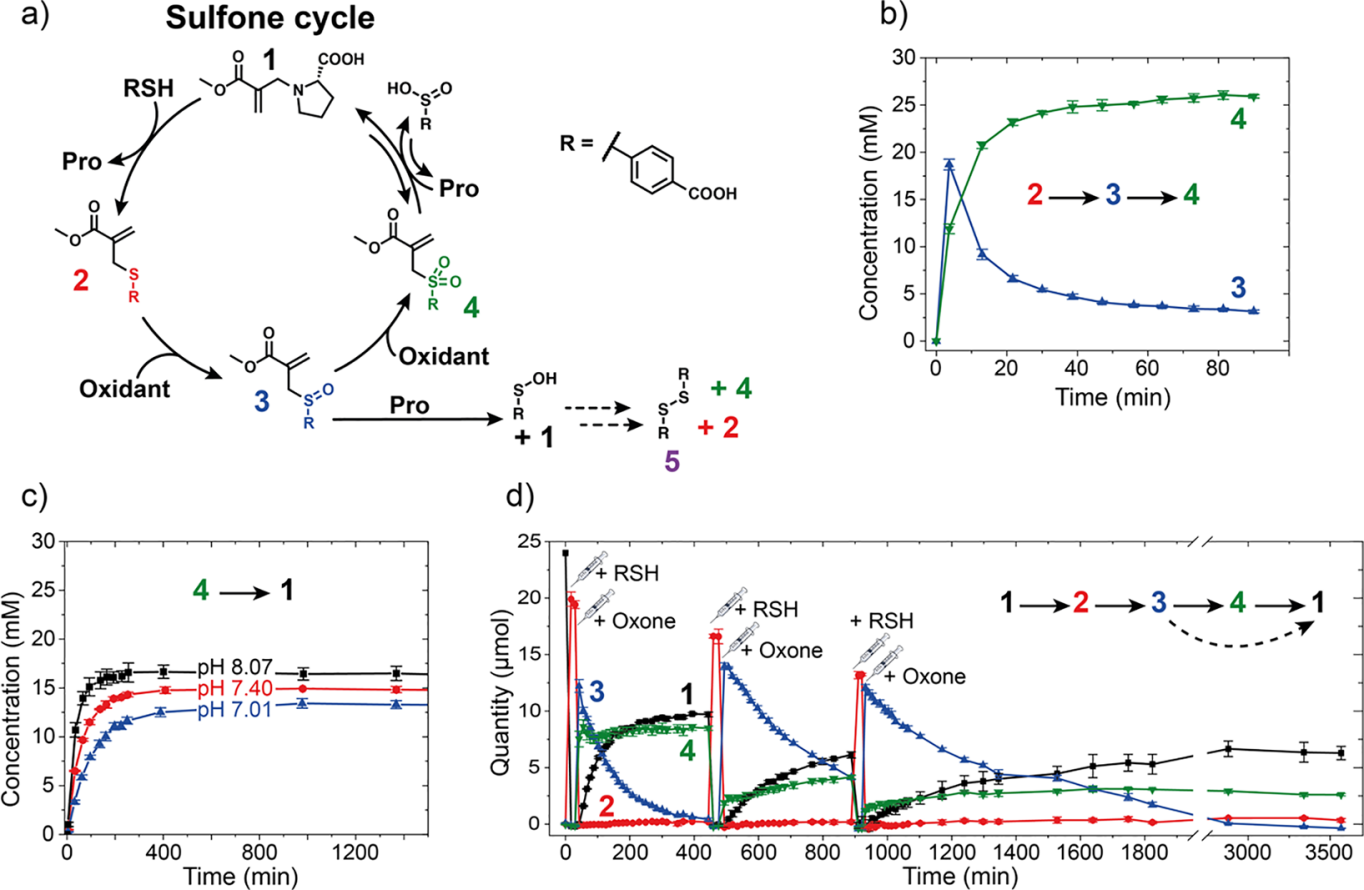
Sulfone reaction network. (a) General scheme of the sulfone reaction network; (b) oxidation of sulfide-MA **2** by oxone at pH 8; (c) proline-MA **1** recovery from the reaction of sulfone-MA **4** with proline at different pHs; (d) stepwise addition of RSH and oxone to proline-MA **1**, the system is re-fueled two times. All kinetic measurements were performed in the same conditions: 10% DMF in 0.5 M phosphate buffer at 20 °C. Error bars represent one standard deviation over three independent experiments.

**Figure 3:**
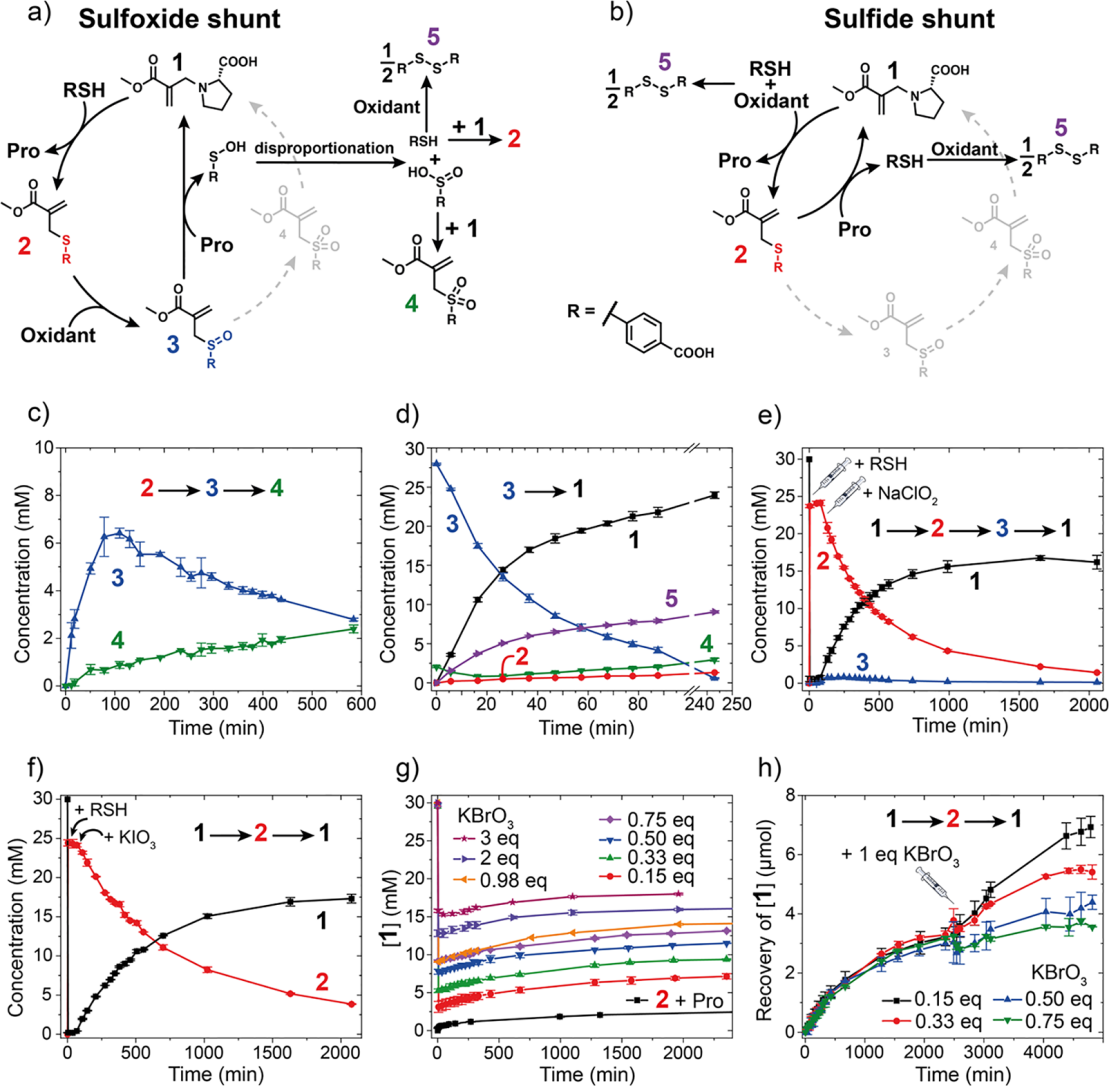
Sulfoxide (a) and sulfide (b) reaction networks. (c) Oxidation of sulfide-MA **2** with KIO_4_; (d) sulfoxide-MA **3** + 1 equiv proline; (e) stepwise addition of RSH and NaClO_2_ to proline-MA **1**; (f) stepwise addition of RSH and KIO_3_ to proline-MA **1**; (g) reaction cycle experiments with different amounts of KBrO_3_; (h) differences in the recovery of proline-MA **1** after addition of another 1 equiv of KBrO_3_ to different reaction cycle experiments. Error bars represent one standard deviation over two (c,e,f,g,h) or three (d) independent experiments.

One of the oxidants of choice to oxidize sulfides to sulfones is oxone (i.e., the complex salt of potassium peroxymonosulfate).^41,42^ As a thiol, we chose water-soluble 4-mercaptobenzoic acid (RSH) due to its high thiol acidity, which makes it both a good nucleophile and stabilized leaving group. We studied the steps of this CRN separately: thiol substitution (**1** → **2**), sulfide oxidation (**2** → **3** → **4**), and sulfone substitution (**4** → **1**) (Figure 2a). The first step (**1** → **2**) proceeds fast and with high yield (typically above 90% in <1 min, Table S2). The sulfide oxidation **2** → **4** proceeds smoothly within 1.5 h via **3** (Figure 2b). Varying the pH from 7.0 to 8.0 does not influence this step (Figure S1, this pH range was found experimentally where the ester of **1** is stable to hydrolysis and nucleophiles react fast with **4**). For the sulfone substitution (**4** → **1**), we studied a range of amines and alcohols (Figures S2–S6) and the stability of the resulting adducts (Figure S7). We found l-proline (Pro) to react fast (*t*_1/2_ = 30 min, at pH 8.0, Figure 2c and Table S2) and with a relatively high yield to proline-MA **1**, which is stable over the observed time. Hence, we decided to proceed with proline as a nucleophile for this CRN. The substitution step **4** → **1** was found to be pH-dependent, proceeding faster and with higher yields at increased pH (pH 7: *t*_1/2_ = 75 min, 45% yield; pH 7.4: *t*_1/2_ = 45 min, 49% yield; pH 8: *t*_1/2_ = 30 min, 56% yield, Figure 2c). This effect is likely due to the increased nucleophilicity of proline at higher pH. As shown in Figure 2c, even at higher pH, the conversions are not quantitative, likely because the substitution step **4** → **1** is an equilibrium reaction. The displaced sulfinate can act as a nucleophile in the reverse reaction **1** → **4** (Figure S8). Having studied all separate CRN steps in detail, we combined them into one system. Starting from a proline-MA **1** solution, we initiated the cycle by adding RSH, followed after 3 to 5 min by the addition of oxone. We found all reactions in the cycle to proceed subsequently, recovering approximately 50% of the original amount of proline-MA **1** (Figure 2d, a line with black squares at 440 min). The system can be run at least three times. After the second addition of thiol and oxidant, significant dampening of the recovery of proline-MA **1** is observed, whereas, after the third addition, proline-MA **1** is recovered at a similar yield as after the second addition although over a longer timescale (Figure 2d, a line with black squares around 3500 min). We found several side reactions that influence the recovery, such as double additions, hydrolysis of substrate **1** over long timescales, and proline oxidation (Scheme S1). Apart from the sulfone substitution (**4** → **1**), proline-MA **1** recovery can also occur via the addition–substitution of proline on sulfoxide-MA **3** (**3** → **1**) (Figure 3d). This reaction will be discussed in detail in the following section. Furthermore, upon adding RSH, a sharp decrease of sulfone-MA **4** and sulfoxide-MA **3** occurs because both species can react with RSH swiftly—being the better nucleophile than proline—recovering sulfide-MA **2** in the process (Figures S9–S11). Contrary to the stepwise addition experiment in Figure 2d, the simultaneous addition of RSH and oxone does not lead to a significant recovery of **1** (Figure S12). This is due to the rapid consumption of RSH by oxone to form RSH-disulfide **5**. Furthermore, oxone can also directly oxidize proline-MA **1** to form oxidized proline-MA and proline to form the corresponding nitrone (Scheme S1 and Figures S13–S15). Despite some off-cycle side reactions when simultaneously adding RSH and oxidant, these results show that with a stepwise manner of addition, we can successfully run the sulfone cycle at least three times, recovering the initial Michael-accepting species mediated by oxidation chemistry, producing the sulfinate and sulfonate of RSH as side products.

### Sulfoxide Shunt

Starting from the observation that proline can also react with sulfoxide-MA **3** (Figure 3d), we hypothesized that the formation of sulfone-MA **4** can be suppressed in favor of **3** using a weaker oxidant, avoiding some of the problematic side reactions of the sulfone cycle (see Scheme S1). This would allow access to a shunt with different yields, timescales, and products in response to the properties of the employed oxidant. We found that oxidants such as hypochlorite and hydrogen peroxide, which are only slightly weaker than oxone (as judged by their oxidation potentials,^43^Figure 1), still yielded sulfone-MA **4**, along with the side reactions associated with this CRN (Figure S16). Potassium periodate, on the other hand, being even weaker, is commonly applied for the selective synthesis of sulfoxides from sulfides and hence should be a good candidate for running the CRN primarily via sulfoxide-MA **3**.^44^ The steps of this CRN were again studied separately. To our surprise, subjecting sulfide-MA **2** to KIO_4_ (oxidation step **2** → **3**) yielded not only sulfoxide-MA **3** but also small amounts of sulfone-MA **4** (Figure 3c). Upon the formation of sulfoxide-MA **3**, we found that, unlike sulfone-MA **4**, **3** is not stable in aqueous media for a prolonged time. Under basic conditions (pH 8), water can attack and displace sulfenic acid (Figure S17). This highly reactive species is responsible for a complex cascade of reactions, which can lead to the formation of sulfide-MA **2**, sulfone-MA **4,** and disulfide **5**, among others (Figure 3a and Scheme S1). This suggests that the sulfone-MA **4** formed in Figure 3c is not due to oxidation with KIO_4_ but due to a degradation reaction of the formed sulfoxide-MA **3** instead. This was further confirmed by subjecting sulfoxide-MA **3** to proline (sulfoxide substitution **3** → **1**, Figure 3d). Even without any oxidant, we found disulfide **5**, sulfide-MA **2,** and sulfone-MA **4** side products forming over time due to the degradation pathways of the sulfenic acid (Figure 3a). Furthermore, we found a higher recovery of proline-MA **1** (∼83%, Figure 3d) compared to the sulfone substitution (∼55%, **4** → **1**, Figure 2c). We achieved similar results for sodium chlorite, which also proceeds primarily through the sulfoxide pathway. Combining stepwise RSH addition and oxidation by NaClO_2_ gave good recovery yields of proline-MA **1** and the transient formation of sulfoxide-MA **3** (Figure 3e). Furthermore, we did not observe the oxidation of proline as we had seen previously with stronger oxidants such as oxone. As expected, when attempting to run this system with the simultaneous addition of RSH and oxidant, we encountered the same challenge as with stronger oxidants: a rapid consumption of RSH to exclusively form disulfide **5**. However, the stepwise addition experiments demonstrate that our CRN can be run via a shunt, mostly avoiding sulfone-MA **4**. Similar to the peroxide shunt of P450 enzymes, where stronger oxidants lead to the degradation of the heme center,^3,45^ the sulfoxide shunt results in a reduced number of side reactions. Unlike the sulfone cycle, the sulfoxide shunt leads to sulfenic acid as the dominant sulfur side product, which disproportionates to produce thiol and sulfinic acid (Figure S17). Thiol can be further oxidized to disulfide **5**, whereas sulfinic acid can react with **1** to produce sulfone-MA **4** (Figure 3a,d).

### Sulfide Shunt

We hypothesized that employing even weaker oxidants might further reduce side reactions and potentially enable us to run the CRN with the simultaneous addition of RSH and oxidant. To our surprise, when testing oxidants such as bromates and iodates, which are too weak to oxidize sulfide-MA **2** to sulfoxide-MA **3** (Figures S18 and S19), we still observed a significant recovery of proline-MA **1** with the stepwise addition of RSH and oxidant, creating a second shunt to the original sulfone cycle (Figures 1c and 3f). While in the first two CRNs (Figure 1a,b), the recovery of **1** is driven by direct oxidation, i.e., activation, of sulfide-MA **2**, in this case, the weaker oxidants remove RSH from the equilibrium between **2** and **1**, forming disulfide **5** as waste (Figure S20) and driving the recovery **2** → **1** forward. While we found similarly high recoveries with KIO_3_ compared to the sulfone CRN (approximately 55%), the timescale of recovery through the sulfide shunt is roughly fivefold longer, 2000 min (Figure 3f and Table S2). This shunt shows even fewer side reactions than the sulfoxide shunt: the only two significant side reactions are double additions of thiol and hydrolysis of substrate **1** over time (Figures S7 and S21 and Scheme S1). Furthermore, when adding RSH and KBrO_3_ to proline-MA **1** simultaneously, we found that, indeed, some recovery of **1** could be observed (approximately 10%), depending on how much oxidant is added (Figure 3g). Adding a large excess of KBrO_3_ leads to lower recoveries as the direct oxidation of RSH to disulfide is favored over its substitution reaction with **1**. Interestingly, we found that the recovery rates are mostly limited by the lifetime of KBrO_3_ in solution as adding an excess of oxidant at a later stage leads to increased recoveries, with the highest recoveries being observed with low initial doses of oxidant (Figure 3h). This effect is because a low initial dose of KBrO_3_ allows more sulfide-MA **2** to form. The sulfide shunt offers yet another pathway for recovery of **1** with fewer side reactions over a longer timescale. Recoveries in the case of stepwise addition are similar to those of the sulfone CRN, while unlike for the sulfone and sulfoxide pathways, in the sulfide shunt, low recovery levels are possible even when RSH and oxidant are added simultaneously. This shunt produces disulfide **5** as the only sulfur side product, avoiding other species and degradation pathways found in the sulfone cycle and sulfoxide shunt. Comparing the kinetics of the three pathways, we found that the sulfone cycle proceeds fastest, with the addition of proline to sulfone-MA **4** to recover **1** as the rate-determining step (Figure 2c and Table S2). In the sulfoxide shunt, oxidation is the rate-determining step. The sulfide shunt is the slowest pathway to recover **1**, with proline-readdition as the rate-determining step (Figure 3e,h). This decrease in kinetics goes hand in hand with fewer side reactions. Interestingly, the yields of the recovery of **1** are similar for each of the available pathways, with the potential to increase the recovery yields with additional portions of oxidant in the sulfide shunt (Figure 3h).

### Reversibility and Recycling Strategy

Metabolic pathways are not always unidirectional.^22,46,47^ The Krebs cycle for example can run in reverse, in the reduction mode, with autotrophic carbon dioxide fixation depending on whether organic or inorganic carbon sources are available.^21,46^ Reversibility is an important aspect of adaptability and is difficult to implement in synthetic systems, especially in redox reactions, as forward and backward processes are usually not orthogonal.

**Figure 4:**
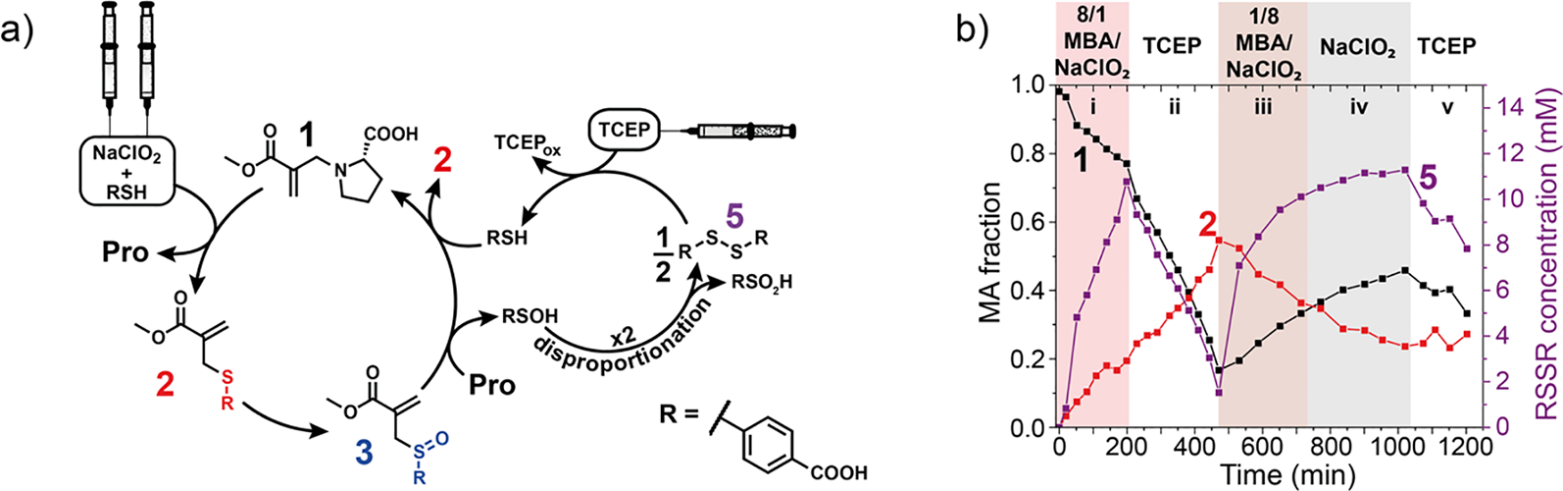
(a) Disulfide recycling with phosphines in the sulfoxide shunt; (b) evolution of proline-MA **1**, sulfide-MA **2**, and disulfide **5** over time under different flow conditions.

In this context, we explored the recovery of RSH from disulfide **5**, which is the major product in both the sulfoxide and sulfide shunts. This allows a partial reversal of the CRN, leading to the formation of sulfide-MA **2** from **1**, driving the main cycle forward again. Phosphines are widely used for disulfide reduction.^48,49^ For this system, we decided to use NaClO_2_ as an oxidant due to its high solubility and stability in water, and tris(2-carboxyethyl)phosphine (TCEP) as a phosphine (Figure 4a). We used a flow setup to efficiently switch the system between an excess of sulfide-MA **2** and an excess of proline-MA **1**. Depending on the flow rate, the apparent reaction rates can be tuned. We start from a solution of proline-MA **1** and explore four different flow regimes. In the first flow regime, (i) an 8/1 excess of thiol relative to oxidant is flowed. This leads to the formation of sulfide-MA **2**, as well as the formation of disulfide **5**, due to the cross-reactivity of the thiol with the oxidant (Figure 4a). In flow regime (ii), both thiol and oxidant flows are stopped and TCEP is flowed. TCEP reduces **5** to free thiol, which leads to the formation of yet more **2**, consuming **1**. In the third phase (iii), an 8/1 excess of oxidant is flowed relative to the thiol. This primarily leads to the oxidation of **2** to form sulfoxide-MA **3**, which can react with free proline to recover **1**. Furthermore, **5** is formed both as a side product from the released sulfenic acid, as well as the direct reaction of thiol and oxidant. In the fourth flow regime (iv), these trends continue, but as only oxidant (NaClO_2_) is flowed, disulfide **5** is exclusively generated as a side product of sulfenic acid degradation. The last flow regime (v) is equal to regime (ii), aiming to reduce disulfide **5** to recover RSH. One of the downsides of using phosphines in this system is that the addition to **1** is possible, yielding phosphine-MA as a side product (Figure S22). These findings show that recycling the major product of the sulfoxide shunt, disulfide **5**, is possible using phosphines, enabling control over CRN speciation under flow conditions over time.

## Conclusions

We introduce the use of shunts in an artificial CRN, offering three distinct pathways toward the recovery of a MA using oxidation chemistry. The oxidant strength dictates if the full CRN or any of the two shunts will preferentially be used without a compromise in recovery yields. The shortest (sulfide) shunt leads to the slowest reaction kinetics and fewest side reactions. Using a flow setup, we can recycle the disulfide product and partially reverse the reaction cycle. Implementing shunts in artificial CRNs will provide more control over chemical speciation and steering of fluxes along different pathways. Our approach mimics nature, where shunts are common to achieve precise control over metabolic processes in response to external and/or internal stimuli. Shunts could help systems chemists to engineer more adaptive CRNs, with possible applications in triggered catalyst release, transient materials, or artificial metabolic networks.

## Supporting Information Available

Synthesis, spectra, supplementary, and control experiments (PDF)
