# Supplementary material for: Redox-Controlled Shunts in a Synthetic Chemical Reaction Cycle
Source: J Am Chem Soc. 2023 Apr 24;145(17):9672–8. doi: 10.1021/jacs.3c00985 (PMC10161229; doi:10.1021/jacs.3c00985)
Supplement: Supplementary file 1 — ja3c00985_si_001.pdf [file ja3c00985_si_001.pdf]

Redox-controlled shunts in a synthetic chemical reaction cycle

Anastasiia Sharko,[a§] Benjamin Spitzbarth,[b§] Thomas M. Hermans,[a*] Rienk Eelkema [b*]

[§] These authors contributed equally.

[a] University of Strasbourg & CNRS, UMR7140, Strasbourg, France.

[b] Department of Chemical Engineering, Delft University of Technology, Van der Maasweg 9, 2629 HZ Delft, The Netherlands.

Supporting Information

Contents

Redox-controlled shunts in a synthetic chemical reaction cycle 1
General Information 2
NMR measurements 2
LC-HRMS measurements 2
Ultimate Scheme: sulfone CRN 3
Synthesis 4
Supplementary experiments 8
T1 measurements 8
Half-lives of individual CRN reactions 8
Sulfide Oxidation at different pH values 10
Sulfone-MA + nucleophiles 10
Sulfone-MA 4 + additional proline: substitution reversibility and the fate of the sulfinate 15
Sulfone-MA 4 + thiol(s) 16
Sulfoxide-MA 3 + thiol 18
Sulfone network: simultaneous addition of MBA and Oxone 19
Oxone: reference reactions 20
Recovery of Proline-MA 1 from Ox-Proline-MA 22
Stepwise addition: comparison of oxidants 23
Sulfoxide-MA stability study 25
Bromate: reference reactions 25
Addition reactions without substitution: Loss of double bond functionality 28
Phosphine + MA 30
References 32
Appendix 34

General Information

Methyl (2-hydroxymethyl)acrylate and L-Proline were purchased from Fluorochem. Methyl (2-bromomethyl)acrylate, Oxone (potassium peroxymonosulfate), and sodium hypochlorite were purchased from TCI Europe. Acetonitrile, DMF, potassium hydrogen phosphate, potassium hydroxide, acetyl chloride, trimethylamine, anhydrous DCM, DSS, sodium chlorite, potassium iodate, potassium periodate, MMPP, SDCI, potassium chlorate, and potassium perchlorate were purchased from Sigma Aldrich. DCM (technical grade) was purchased from VWR International. D_2O, MeOD, $CDCl_3$, and DMSO-d_6 were purchased from Eurisotop. Deionized (milliQ) water was made in our laboratory. Unless stated otherwise, all chemicals were used as received. For water-free experiments, anhydrous solvents and pre-dried flasks were used.

NMR measurements

NMR spectra were recorded on an Agilent-400 MR DD2 (400 MHz for ^{1}H, 101 MHz for ^{13}C) instrument. All measurements were taken at 298 K. For qNMR measurements, sealed ampoules (short NMR tubes with an outer diameter of 3 mm, purchased from VWR International) were prepared, containing a 10 mM solution of sodium trimethylsilylpropanesulfonate (DSS) in D_2O to ensure i) no potential reaction between standard and sample and ii) no presence of deuterated solvent in the sample to avoid a change in kinetics due to isotope effects. **All qNMR measurements were conducted in a 9/1 mixture of 0.5 M pH 8.0 potassium phosphate buffer and DMF unless stated otherwise.** For data analysis, the raw data was treated in Mestrenova V11.0. The 0.0 ppm signal of DSS was integrated and set to 1000, and the sample concentrations were derived accordingly based on the known starting concentrations. To ensure full relaxation of all protons, T1 measurements (see Table S1) were performed, and the scan time was set to 5xT1 for the slowest relaxing signal in each experiment, respectively. All kinetic studies were performed using PRESAT experiments with 8 scans and suppression of the H_2O peak.

LC-HRMS measurements

LC-HRMS was performed with a Thermofisher Scientific UltiMate 3000 RSLCnano UHPLC System coupled with EMT Thermo OrbiTrap Mass analyzer. The ionization used was ESI using a Hypersil GOLD column, 50 x 2.1 mm, 1.9 µm.

Ultimate Scheme: sulfone CRN

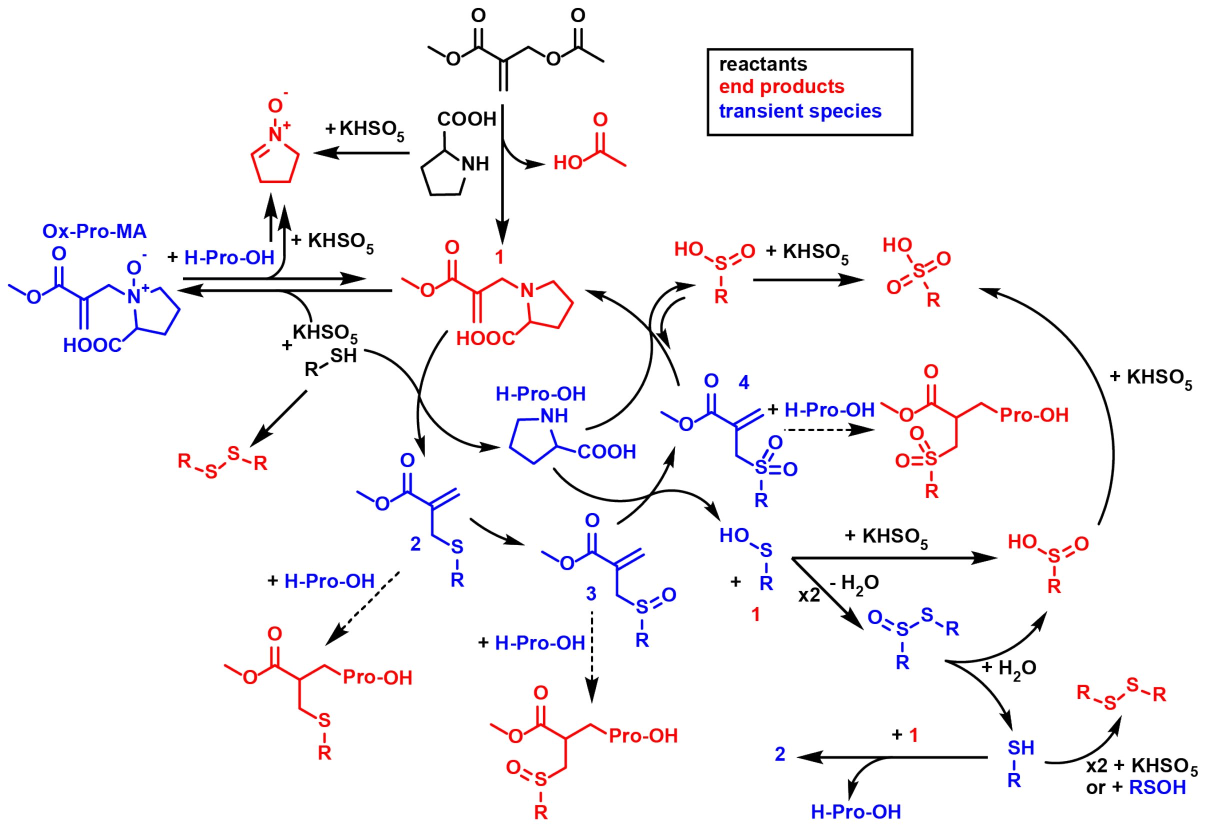

Scheme S1: Main reaction network of sulfone CRN, starting from proline-MA, followed by the addition of thiol (MBA) and Oxone. All identified side reactions are shown. For clarity, starting materials are colored black, species that form transiently are colored blue, and species that are present at the equilibrium after consumption of all reagents are colored red.

In the following, a chronological explanation of the reactions and side reactions we identified in the sulfone CRN (main text, Figure 1, left) follows. We chose to explain this CRN in detail, as it contains all identified reactions—the pathway and (side) reactions of the sulfoxide CRN (main text, Figure 1, center) and the sulfide CRN (main text, Figure 1, right) present a subset of the (side) reactions shown here.

The CRN starts by preformation of proline-MA **1** via the addition and substitution of proline to AcO-MA, expelling acetate, which is inert under our conditions (see synthesis section for preparation of stock solution). (**Note:** an avoidance of methyl-2-(bromomethyl)acrylate (Br-MA) is crucial here due to the formation of hypobromite and bromine from the reaction of bromide with Oxone,[1] the latter of which we also confirmed visually as a brown vapor forming.) Next, thiol (MBA) and Oxone are added, upon which several reactions can occur.

First, on the CRN's main route, thiol can react with proline-MA **1**, expelling proline as a transient species under the formation of sulfide-MA **2**. Furthermore, all three species involved in this first step (proline-MA **1**, thiol, and proline) can also react with Oxone in unwanted side reactions. This leads to the following:

i) Direct oxidation of proline, first to *N*-hydroxyproline,[2] and subsequently under decarboxylation to the corresponding nitrone as shown in literature.[3] We observed the characteristic double bond signal of said nitrone (see Figure S13).

ii) Oxidation of proline-MA to oxidized proline-MA (Ox-Proline-MA in Scheme S1) which can degrade under double addition or re-form proline-MA **1** via substitution with proline under release of *N*-hydroxyproline (Figure S15).

iii) Oxidation of free thiol to form disulfide and subsequently higher oxidized species.

Continuing on the main route of the CRN, the remaining Oxone can now oxidize sulfide-MA **2** to sulfoxide-MA **3** and sulfone-MA **4**. Oxone is a capable reagent for this task, as it can efficiently oxidize sulfides to sulfones, leaving the allylic double bond untouched, as previously shown in literature.[4] While Oxone can generally oxidize alkenes, this is only possible with electron-rich alkenes in the presence of ketones.[5] One of the minor side reactions that can occur at each stage of sulfide-MA **2**, sulfoxide-MA **3,** and sulfone-MA **4** is the addition of proline under loss of double bond functionality. The double adduct species were identified by LC-HRMS.

At the stage of sulfoxide-MA **3**, proline can now undergo an addition-substitution reaction, expelling the corresponding sulfenic acid under the generation of starting proline-MA **1**. This step is irreversible, as sulfenic acids are notorious for their high reactivity,[6] leading to their swift degradation. They can either be directly oxidized to sulfinates (and sulfonates subsequently) or dimerize under the release of water to form thiosulfinates. These thiosulfinates can either comproportionate with sulfinates to form thiosulfonates, or, more likely under our conditions, hydrolyze (under sulfide-catalysis) to yield a thiol and a sulfinate, as shown in literature.[6,7] The thiol formed in this process can either be oxidized by free Oxone or react with proline-MA **1** to generate more sulfide-MA **2**. We confirmed these pathways by reacting sulfoxide-MA **3** with proline and identifying the proposed side products (Figure 3d, main text).

At the last stage of the main route, sulfone-MA **4** is present and can directly react with proline to recover proline-MA **1** under the generation of free sulfinate. This last step has been shown before in literature for the functionalization of lysine residues.[8] We propose that this reaction is an equilibrium (see Figure S8) and will only lead to high yields if the released sulfinate is further oxidized to inert sulfonate, which presents the final waste species.

The cycle is closed, and the recovered proline-MA **1** can now undergo the same reactions again upon the addition of new portions of thiol and oxidant (see repeated oxidant additions in Figure 2d, main text).

Synthesis

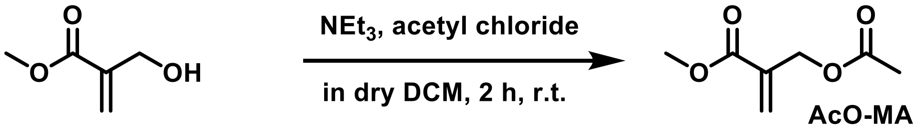

Methyl (2-acetoxymethyl)acrylate (AcO-MA) was synthesized according to a literature procedure.[9] Briefly, 1.161 g (1.030 mL, 10.0 mmol, 1.0 eq.) methyl (2-

hydroxymethyl)acrylate were dissolved in 20 mL anhydrous DCM under argon. The solution was cooled to 0 °C in an ice bath. 1.113 g (1.525 mL, 11.0 mmol, 1.1 eq.) triethylamine were added, followed by the dropwise addition of 0.785 g (0.714 mL, 10.0 mmol, 1.0 eq.) acetyl chloride. The ice bath was removed, and a colorless precipitate formed within approximately one minute. After stirring at r.t. for two hours, the mixture was filtered, and the filter was rinsed with additional DCM. The organic phase was washed with 20 mL water twice and with 20 mL brine once. The organic phase was dried over $MgSO_4$, and the solvent was removed under reduced pressure. The crude, yellow liquid was purified via a short silica plug (gradient 10/1 → 8/1 PE/EA) to yield 1.32 g (8.5 mmol, 85%) as a colorless oil.

^{1}H-NMR (400 MHz, $CDCl_3$): δ = 6.36 (s, 1H, C=C*H*$_2$), 5.84 (s, 1H, C=C*H*$_2$), 4.80 (s, 2H, C*H*$_2$OAc), 3.78 (s, 3H, OC*H*$_3$), 2.10 (s, 3H, O(O)CC*H*$_3$). The spectroscopic data was found to be in accordance with the literature data.[9]

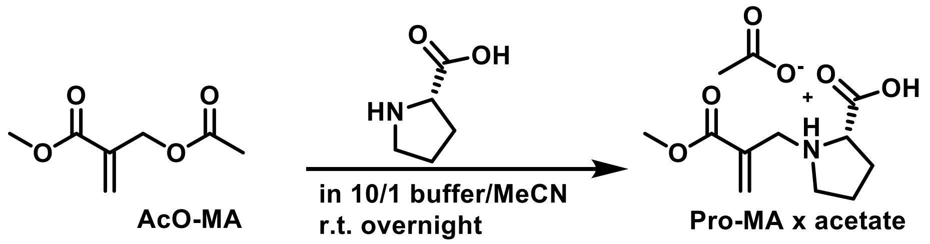

Note: This synthesis proved to be challenging. A synthesis starting from Br-MA was not feasible as the product would contain bromide as a counterion which can react with some oxidants, forming oxidized bromine species. The bromide counterion proved difficult to remove. Hence, a switch to AcO-MA was necessary to introduce the inert acetate counterion. Also, using a buffer is crucial to avoid acidification from the release of acetic acid over time which would halt the reaction by protonating free proline. Furthermore, the pH MUST NOT exceed 8.0 as this will lead to swift hydrolysis of the methyl ester moiety. Hence, the use of other strong, sacrificial bases instead of the buffer is also not feasible. The product was not isolated from the buffer salts and was used as a stock solution, the concentration of which was determined by adding a known amount of pure 4-mercaptobenzoic acid (MBA) and comparing the signals of remaining Proline-MA and sulfide-MA.

(2-(methoxycarbonyl)allyl)-L-proline (proline-MA **1**) was synthesized by dissolving 229.30 mg (2.0 mmol, 1.0 eq.) L-proline in a mixture of 10 mL 0.5 M pH 7.4 potassium phosphate buffer and 1 mL acetonitrile. (**Note:** acetonitrile is not necessary, however, it speeds up the reaction by aiding the dissolution of hydrophobic AcO-MA.) 647.6 mg (585.0 µL, 2.4 mmol, 1.2 eq.) AcO-MA were added dropwise. The mixture was vigorously stirred at room temperature overnight, upon which the suspension cleared up. The excess AcO-MA was removed by extracting with 15 mL EtOAc twice. The acetonitrile was removed under reduced pressure, and the water was removed by lyophilizing. The resulting colorless powder was dissolved in 9/1 0.5 M potassium phosphate buffer/DMF (pH dependent on the experiment) to yield a 70 mM stock solution. The yield, determined by NMR, usually exceeded 90 %, depending on the batch. **Note:** The following spectra were taken of a batch without acetate as a counterion.

^{1}H-NMR (400 MHz, MeOD): δ = 6.56 (s, 1H, $\mathrm{C{=}C}\mathit{H}_2$), 6.28 (s, 1H, $\mathrm{C{=}C}\mathit{H}_2$), 4.20 – 4.08 (m, 2H, $\mathrm{C_qC}\mathit{H}_2\mathrm{N}$, **Note:** we found that the observed multiplicity of these CH_2 signals strongly depends on the solvent.), 3.93 (ddd, J = 9.6, 5.2, 1.7 Hz, 1H, $\mathrm{NC}\mathit{H}\mathrm{COOH}$), 3.83 (s, 3H, $\mathrm{C(O)OC}\mathit{H}_3$), 3.72 (ddd, J = 11.8, 8.3, 5.1 Hz, 1H, $\mathrm{NC}\mathit{H}_2\mathrm{CH_2}$), 3.26 – 3.14 (m, 1H, $\mathrm{NC}\mathit{H}_2\mathrm{CH_2}$), 2.52 – 2.38 (m, 1H, $\mathrm{NCH(COOH)C}\mathit{H}_2$), 2.22 – 2.04 (m, 2H, $\mathrm{NCH(COOH)C}\mathit{H}_2$, $\mathrm{NCH_2C}\mathit{H}_2$), 1.94 (dq, J = 13.2, 8.5 Hz, 1H, $\mathrm{NCH_2C}\mathit{H}_2$).

^{13}C-NMR (101 MHz, MeOD): δ = 173.13 (COOH), 167.05 (COOMe), 135.58 ($\mathrm{C_q{=}}\mathit{C}\mathrm{H_2}$), 132.74 ($\mathit{C}_\mathrm{q}\mathrm{{=}CH_2}$), 70.64 ($\mathrm{N}\mathit{C}\mathrm{HCOOH}$), 56.61 ($\mathrm{C_q}\mathit{C}\mathrm{H_2N}$), 55.99 ($\mathrm{N}\mathit{C}\mathrm{H_2CH_2}$), 53.25 ($\mathrm{C(O)O}\mathit{C}\mathrm{H_3}$), 30.18 ($\mathrm{NCH(COOH)}\mathit{C}\mathrm{H_2}$), 24.64 ($\mathrm{NCH_2}\mathit{C}\mathrm{H_2}$).

ESI-LC/HRMS (m/z): calculated for $[C_{10}H_{16}NO_4]^+$: 214.1074, found: 214.1065; calculated for $[C_{10}H_{14}NO_4]^-$: 212.0928, found: 212.0923.

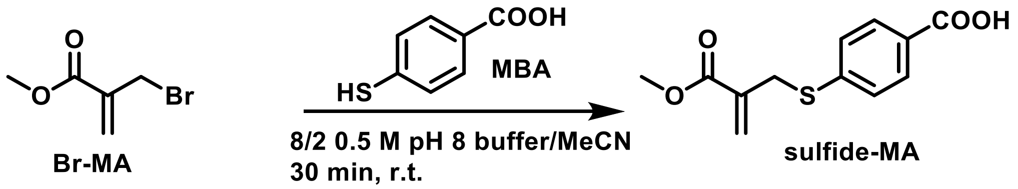

4-((2-(methoxycarbonyl)allyl)thio)benzoic acid (sulfide-MA **2**) was synthesized by dissolving 154.18 mg (1.0 mmol, 1.0 eq.) 4-mercapto benzoic acid (MBA) in 10 mL of an 8/2 mixture of 0.5 M pH 8 potassium phosphate buffer and acetonitrile. 179.01 mg (119.34 µL, 1.0 mmol, 1.0 eq.) of Br-MA were added dropwise. After stirring at room temperature for 30 minutes, the acetonitrile was mostly removed under reduced pressure, upon which the solution turned cloudy. (**Note:** Unlike sulfoxide- and sulfone-MA, sulfide-MA is not soluble in pure buffer even at elevated pH. Hence, it is important to add the reactants in a 1:1 ratio as precisely as possible, as washing the aqueous phase will extract excess Br-MA and sulfide-MA even before acidification.) The aqueous phase was then diluted with approximately 10 mL water, acidified to pH 1 with approximately 5 mL 1 M HCl, and extracted with 20 mL EtOAc thrice. The combined organic phases were dried over $MgSO_4$, and the solvent was removed under reduced pressure to yield 227.1 mg (0.9 mmol, 90%) sulfide-MA **2** as a pale-yellow powder.

^{1}H-NMR (400 MHz, MeOD): δ = 7.95 – 7.87 (m, 2H, $\mathrm{C}\mathit{H}\mathrm{C_qCOOH}$), 7.41 – 7.35 (m, 2H, $\mathrm{C}\mathit{H}\mathrm{C_qS}$), 6.17 (s, 1H, $\mathrm{C{=}C}\mathit{H}_2$), 5.76 (s, 1H, $\mathrm{C{=}C}\mathit{H}_2$), 3.92 (s, 2H, $\mathrm{C}\mathit{H}_2\mathrm{S}$), 3.77 (s, 3H, $\mathrm{C(O)OC}\mathit{H}_3$).

^{13}C-NMR (101 MHz, MeOD): δ = 169.39 (COOH), 167.85 (COOMe), 144.02 ($\mathit{C}_\mathrm{q}\mathrm{COOH}$), 137.52 ($\mathit{C}_\mathrm{q}\mathrm{S}$), 131.16 ($\mathit{C}\mathrm{HC_qCOOH}$), 129.33 ($\mathit{C}_\mathrm{q}\mathrm{{=}CH_2}$), 129.12 ($\mathit{C}\mathrm{HC_qS}$), 127.74 ($\mathrm{C_q{=}}\mathit{C}\mathrm{H_2}$), 52.62 ($\mathit{C}$OOMe), 34.55 ($\mathit{C}\mathrm{H_2S}$).

ESI-LC/HRMS (m/z): calculated for $[C_{12}H_{11}O_4S]^-$: 251.0384, found: 251.0382.

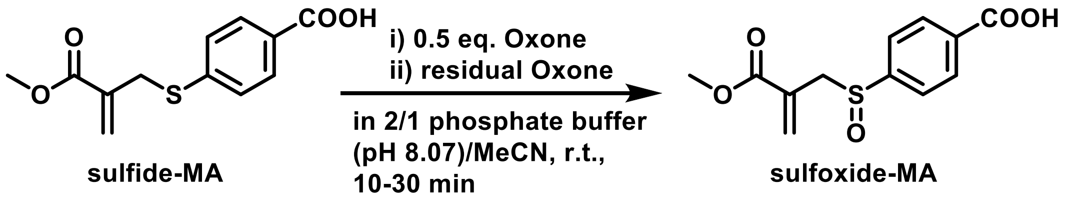

4-((2-(methoxycarbonyl)allyl)sulfinyl)benzoic acid (sulfoxide-MA **3**) was synthesized by dissolving 47.58 mg (0.189 mmol, 1.0 eq.) sulfide-MA in 6 mL of a 2/1 mixture of 0.5 M pH 8.07 potassium phosphate buffer and acetonitrile. 28.97 mg (94.25 µmol, 0.5 eq.) Oxone was dissolved in 1 mL of water and added to the MA-sulfide solution. The solution was stirred at room temperature for 10 minutes. Upon NMR measurement, it was determined that 44.6 % of sulfide-MA had reacted to sulfoxide-MA. Hence, it was calculated that 35.2 mg Oxone were needed to achieve full conversion. This amount was weighed out, added as a solution in 1 mL water, and it was stirred for a further 30 minutes. After completion, the reaction mixture was diluted with 15 mL water and acidified to pH 1 with approximately 5 mL 1 M HCl. Upon acidification, a thick, colorless precipitate formed. The milky suspension was extracted with 20 mL chloroform thrice. The combined organic layers were washed with 10 mL 1 M HCl and dried over $MgSO_4$. The solvent was removed under reduced pressure to yield 42.38 mg (0.158 mmol, 84%) of sulfoxide-MA **3** as a colorless powder. **Note:** Unlike sulfide-MA **2** and sulfone-MA **4**, which we found to be stable over months at room temperature, sulfoxide-MA **3** tends to degrade and needs to be stored in the fridge or preferably the freezer and be used up within several days.

^{1}H-NMR (400 MHz, MeOD): δ = 8.22 – 8.16 (m, 2H, C*H*C$_q$COOH), 7.76 – 7.71 (m, 2H, C*H*C$_q$SO), 6.43 (d, J = 0.9 Hz, 1H, C=C*H*$_2$), 5.78 – 5.76 (m, 1H, C=C*H*$_2$), 4.02 (dd, J = 12.9, 1.0 Hz, 1H, C*H*$_2$SO, **Note:** we found that the multiplicity of these CH_2 protons strongly depends on the solvent.), 3.91 (dd, J = 12.9, 0.8 Hz, 1H, C*H*$_2$SO, **Note:** we found that the multiplicity of these CH_2 protons strongly depends on the solvent.), 3.62 (s, 3H, C(O)OC*H*$_3$).

^{13}C-NMR (101 MHz, MeOD): δ = 168.43 (*C*OOH), 167.17 (*C*OOMe), 148.22 (*C*$_q$COOH), 135.09 (*C*$_q$SO), 133.45 (C$_q$=*C*H$_2$), 131.36 (*C*HC$_q$COOH), 130.20 (*C*$_q$=CH$_2$), 125.79 (*C*HC$_q$SO), 59.08 (*C*H$_2$SO), 52.73 (C(O)O*C*H$_3$).

ESI-LC/HRMS (m/z): calculated for $[C_{12}H_{11}O_5S]^-$: 267.0333, found: 267.0333; calculated for $[C_{12}H_{13}O_5S]^+$: 269.0478, found: 269.0468.

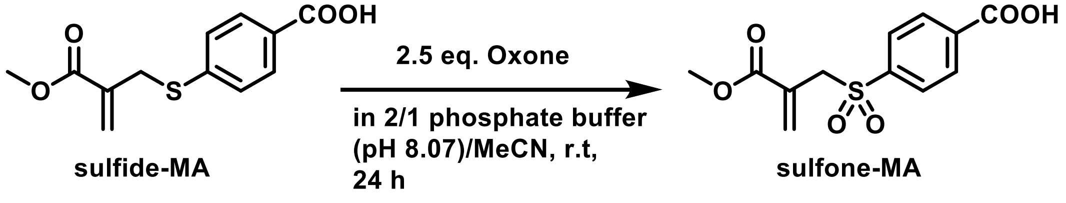

4-((2-(methoxycarbonyl)allyl)sulfonyl)benzoic acid (sulfone-MA **4**) was synthesized by dissolving 47.58 mg (0.189 mmol, 1.0 eq.) sulfide-MA in 6 mL of a 2/1 mixture of 0.5 M pH 8.07 potassium phosphate buffer and acetonitrile. 144.85 mg (0.471 mmol, 2.5 eq.) Oxone were dissolved in 1 mL of water and added to the MA-sulfide solution. A further 2 mL water were added to dissolve the Oxone fully. The solution was stirred at room temperature for

24 hours. After completion, the reaction mixture was diluted with 15 mL water and acidified to pH 1 with approximately 5 mL 1 M HCl. Upon acidification, a thick, colorless precipitate formed. The milky suspension was extracted with 20 mL chloroform thrice. The combined organic layers were washed with 10 mL 1 M HCl and dried over $MgSO_4$. The solvent was removed under reduced pressure to yield 40.68 mg (0.143 mmol, 76%) of sulfone-MA **4** as a colorless powder.

^{1}H-NMR (400 MHz, MeOD): δ = 8.23 (d, J = 8.0, 2H, C*H*C$_q$COOH), 7.96 (d, J = 8.0, 2H, C*H*C$_q$SO$_2$), 6.45 (s, 1H, C=C*H*$_2$), 5.85 (s, 1H, C=C*H*$_2$), 4.33 (s, 2H, C*H*$_2$SO$_2$), 3.60 (s, 3H, C(O)OC*H*$_3$).

^{13}C-NMR (101 MHz, MeOD): δ = 167.88 (*C*OOH), 166.80 (*C*OOMe), 143.40 (*C*$_q$COOH), 137.19 (*C*$_q$SO$_2$), 134.41 (C$_q$=*C*H$_2$), 131.37 (*C*HC$_q$COOH), 130.59 (*C*$_q$=CH$_2$), 130.05 (*C*HC$_q$SO$_2$), 58.28 (*C*H$_2$SO$_2$), 52.83 (C(O)O*C*H$_3$).

ESI-LC/HRMS (m/z): calculated for $[C_{12}H_{11}O_6S]^-$: 283.0282, found: 283.0282; calculated for $[C_{12}H_{13}O_6S]^+$: 285.0427, found: 284.0417.

Supplementary experiments

T1 measurements

To allow for qNMR measurements to be conducted, we measured the T1 times for compounds to be quantified via inversion-recovery experiments. Measurements were performed under the same conditions (i.e. solvent, concentration, and temperature) as in the kinetic studies. T1 times were extracted for the double bond signals of MA species and can be found tabulated below.

Table S1: Compounds that were used in qNMR, the signal of the nucleus that was used for quantification, and its calculated T1 time.

Compound	Signal (ppm)	T1 time (s)
Sulfone-MA **4**	6.35	0.85
Sulfoxide-MA **3**	6.25	0.80
Proline-MA **1**	7.40	1.04
Sulfide-MA **2**	5.93	1.40
TCEP-MA adduct	6.45	0.73
Disulfide **5**	7.62	1.96
Morpholine-MA	6.22	1.09
Piperidine-MA	6.53	0.99
Lysine-MA	5.92	1.45
4-amino benzoic acid-MA	5.65	0.74
ATMA-MA	6.09	0.86
N-methyl taurine-MA	6.22	0.92
Aspartic acid-MA	6.27	0.82

Half-lives of individual CRN reactions

We have extracted the apparent half-lives from the graphs of separately studied reactions wherever possible. Although the half-lives of these individually studied reactions do not

necessarily reflect the kinetics of the whole network, they are convenient to compare experiments with each other.

Comparing the proline reaction with sulfone-MA **4**, sulfoxide-MA **3**, and sulfide-MA **2** separate from the full network (Proline-MA **1** formation in entries # 2, 3, and 4), one can see that reaction with sulfoxide-MA **3** is slightly faster than with sulfone-MA **4** (20 min vs. 28 min). It is the slowest with sulfide-MA **4** (413 min).

The recovery rate of proline-MA **1** is increased in the reaction with sulfide-MA **4** by adding an oxidant KIO_3 (329 min, entry # 7).

Proline-MA **1** recovery in separate steps is faster than within the full network (28 min vs. 31 min, entries # 2, 5) and sulfoxide shunt (20 min vs. 202 min, entries # 3, 6).

Proline-MA **1** recovery is faster in the full cycle (via the sulfone, 31 min, entry # 5) than in the sulfoxide shunt (202 min, entry # 6) and the sulfide shunt (329 min, entry # 7).

Finally, in the sulfide shunt, adding thiol and oxidant simultaneously or stepwise also influences the rate of proline-MA **1** recovery (643 vs. 329 min).

Table S2: Half-lives of different reactions of the whole CRN and the two shunts, determined from ^{1}H-NMR experiments, with standard deviations.

#		Reaction	Half-life, $t_{1/2}$, min
1	Sulfide oxidation	Sulfide-MA **2** oxidation with 2.5 eq. Oxone at pH 8	Sulfoxide-MA **3** consumption 9.3±0.5 min<br>Sulfone-MA **4** formation 4.9±0.2 min
2	Sulfone + Pro	Sulfone-MA **4** + 1.0 eq. Proline at pH 8	Sulfone-MA **4** consumption 26±1 min<br>Proline-MA **1** formation 28±1 min
3	Sulfoxide + Pro	Sulfoxide-MA **3** + 1.0 eq. Proline at pH 8	Sulfoxide-MA **3** consumption 25±4 min<br>Proline-MA **1** formation 20±3 min
4	Sulfide + Pro	Sulfide-MA **2** + 1.0 eq. Proline at pH 8	Sulfide-MA **2** consumption 1480±172 min<br>Proline-MA **1** formation 413±100 min
5	Sulfone cycle stepwise	Sulfide-MA **2** + 1.0 eq. Proline + 2.5 eq. Oxone at pH 8	Proline-MA **1** formation 31±1 min
6	Sulfoxide cycle stepwise	Sulfide-MA **2** + 1.0 eq. Proline + 2.5 eq. $NaClO_2$ at pH 8	Sulfide-MA **2** consumption 283±10min<br>Proline-MA **1** formation 202±7 min
7	Sulfide cycle stepwise	Sulfide-MA **2** + 1.0 eq. Proline + 2.5 eq. KIO_3 at pH 8	Sulfide-MA **2** consumption 541±1min<br>Proline-MA **1** formation 329±29 min

8	Sulfide cycle simultaneous addition	Sulfide-MA **2** + 1.0 eq. Proline + 0.33 eq. $KBrO_3$ at pH 8	Sulfide-MA **2** consumption 941±11 min Proline-MA **1** formation 643±7 min

Sulfide Oxidation at different pH values

We tested the oxidation of sulfide-MA **2** to sulfone-MA **4** with 2.5 equivalents of Oxone at different pH values (Figure S1). Firstly, it is apparent that the oxidation happens via sulfoxide-MA **3** and that this step is relatively fast, showing the highest conversions within several minutes. Sulfoxide-MA **3** then gets further oxidized to sulfone-MA **4** within roughly one hour. Furthermore, in the pH range we tested, we did not see a significant impact of the pH value on the oxidation rates. This is plausible, as the HSO_5^- anion, which is the most abundant form at this pH value, has a pK_a value of 9.88. Hence, the SO_5^{2-} form with a lower oxidation potential only becomes the dominant species at pH values above 10.[10]

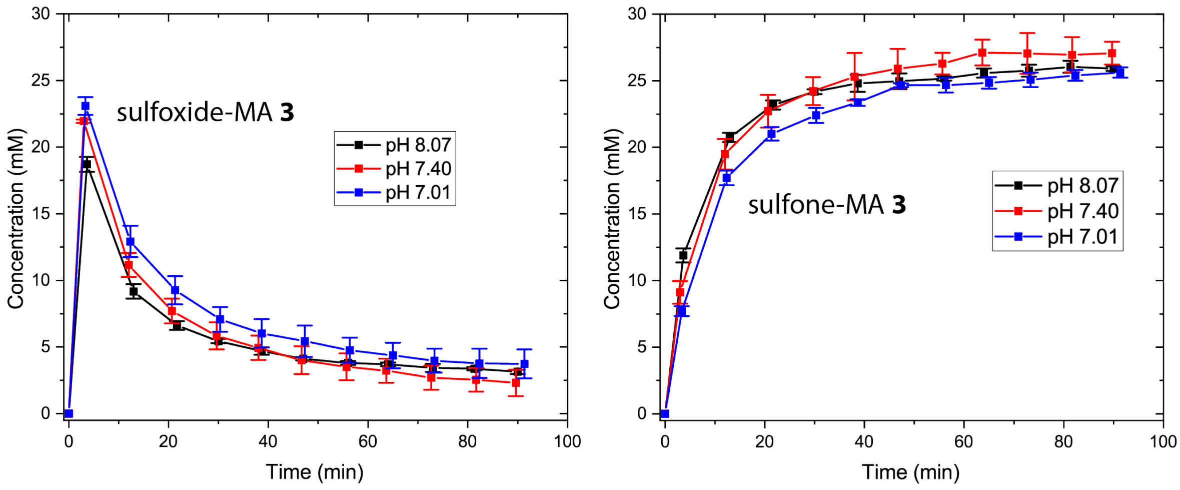

Figure S1: The oxidation of sulfide-MA **2** to sulfone-MA **4** via sulfoxide-MA **3** in the presence of 2.5 eq. of Oxone.

Sulfone-MA + nucleophiles

To determine which nucleophile is most suitable to run the addition-substitution reaction on sulfone-MA **4**, we tested a range of primary and secondary amines as these species are most likely to be able to undergo this reaction (Figure S2). We found that, in general, amines, both primary and secondary, react well with sulfone-MA **4**. Especially for morpholine, we found swift, high conversions (red line), however, the product is not stable over time. We found similar results for the reaction of sulfone-MA **4** with *N*-methyltaurine, piperidine, and (2-aminoethyl)trimethylammonium chloride hydrochloride (ATMA), although their degradation was somewhat slower than that of the morpholine-MA adduct. The addition with 3-aminobenzoic acid also proceeds smoothly, although over a relatively long timescale with lower conversions. Hence, we found that proline is the best nucleophile candidate due to the

relatively fast product formation and stability over time (dark grey line with squares, Figure S2, Figure S7).

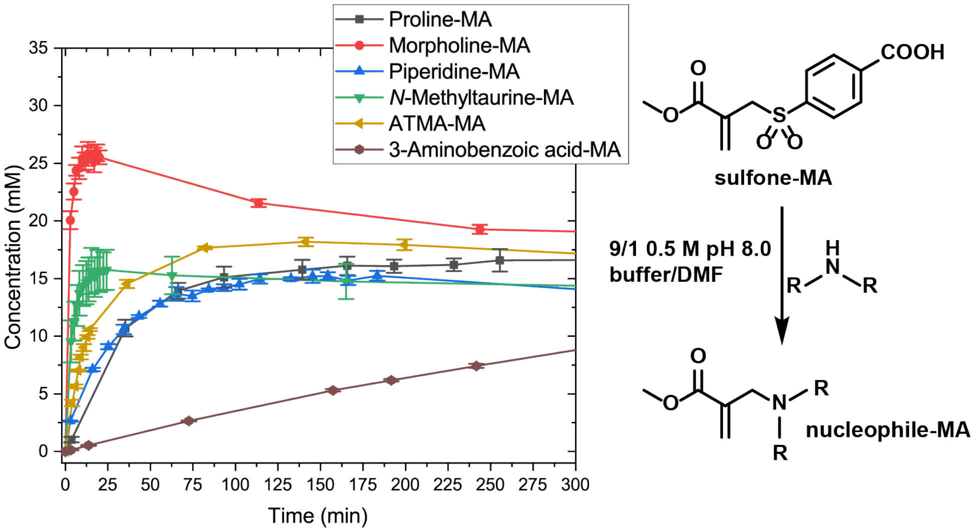

Figure S2: Reaction of different amine nucleophiles with sulfone-MA **4** to yield nucleophile-substituted MAs.

Apart from the nucleophiles that can substitute on sulfone-MA **4** discussed above, we tested several other nucleophiles. We found that phenol, tertiary amines such as trimethylamine, and pyrroles such as pyrrole-2-carboxylic acid do not react with sulfone-MA. It has been shown in the literature that aliphatic alcohols can substitute sulfone MAs under certain conditions (in the presence of K_2CO_3, see compound 5, condition iii. in reference).[11] Our rationale was that phenols, which are more acidic than aliphatic alcohols, should be able to act as nucleophiles under our conditions as well. However, no substitution took place (see Figure S3). Furthermore, in accordance with the literature, we found that tertiary amines cannot substitute on sulfone-MA (Figure S4) due to the lack of the hydrogen-bonding motif.[8] Pyrrole-2-carboxylic acid can likely not participate in a substitution reaction due to the nitrogen's free electron pair being involved in aromaticity (Figure S5). We also tested the reaction of several other nucleophiles that successfully substituted on sulfone-MA. The conversion plots for these nucleophiles were left out in the main text for clarity reasons and the presence of some side reactions that will be discussed below.

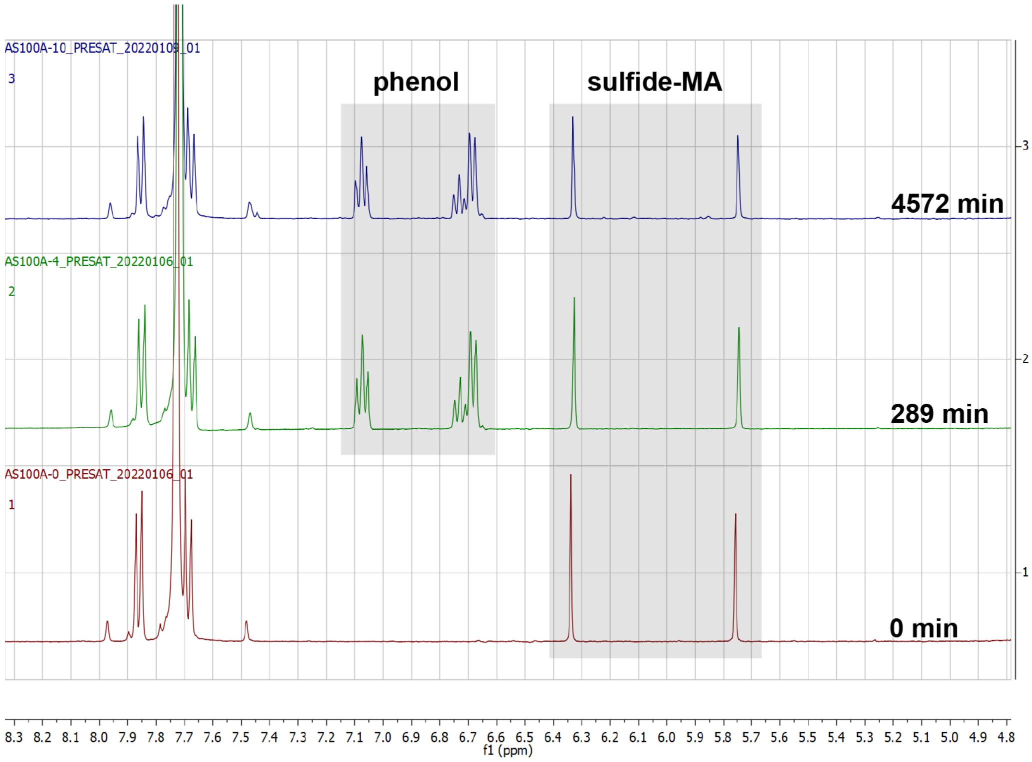

Figure S3: ^{1}H-NMR cutout; bottom: sulfide-MA **2** reference; middle and top: sulfide-MA **2** after addition of 1.0 eq. phenol. No reaction could be observed. As stated in the general section, standard NMR conditions were used (i.e. 9/1 0.5 M pH 8.0 phosphate buffer/DMF) unless stated otherwise.

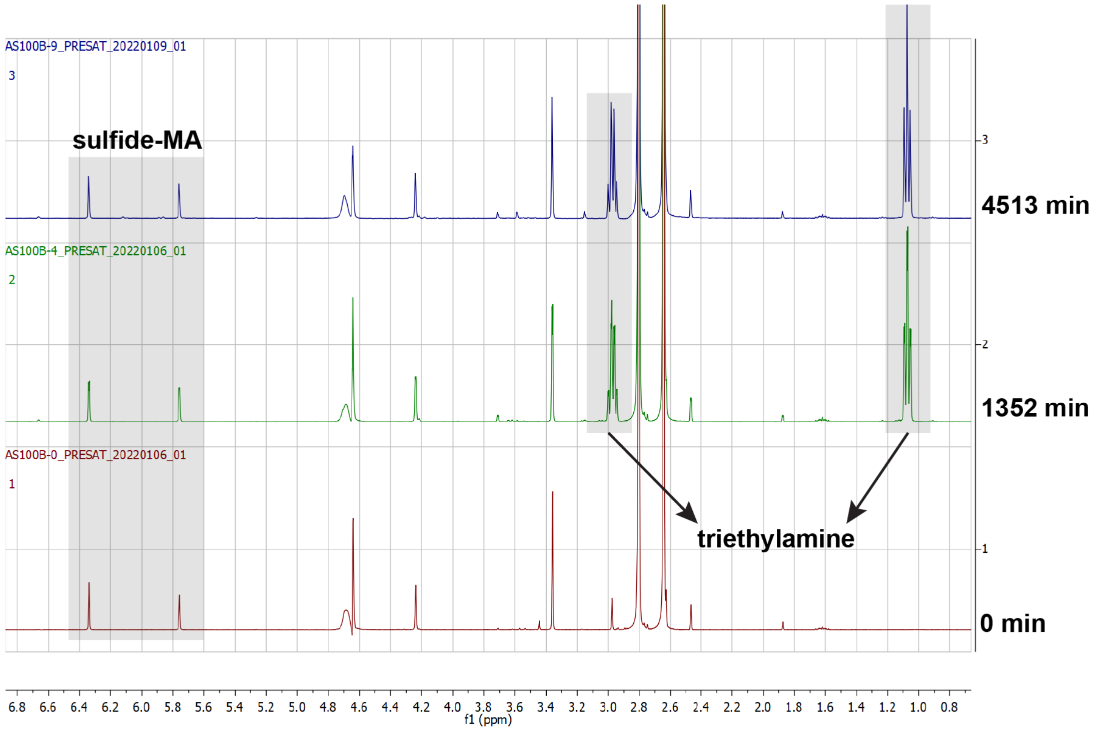

Figure S4: ^{1}H-NMR cutout; bottom: sulfide-MA **2** reference; middle and top: sulfide-MA **2** after addition of 1.0 eq. triethylamine. No reaction could be observed.

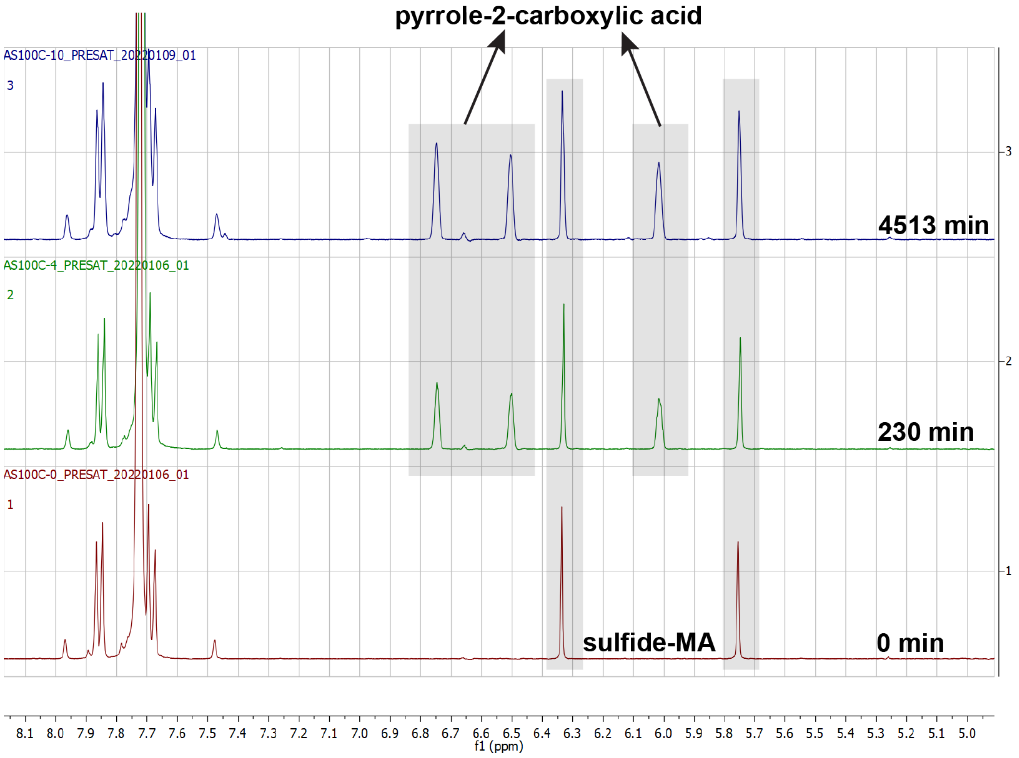

Figure S5: ^{1}H-NMR cutout; bottom: sulfide-MA **2** reference; middle and top: sulfide-MA **2** after addition of 1.0 eq. pyrrole-2-carboxylic acid. No reaction could be observed.

The reaction of sulfone-MA **4** with the primary amines aspartic acid and *N*-acetyl lysine (Figure S6) showed the formation of double addition products, which made these species not suitable. Interestingly, the third primary amine we tested, ATMA, did not show this double addition (Figure S2).

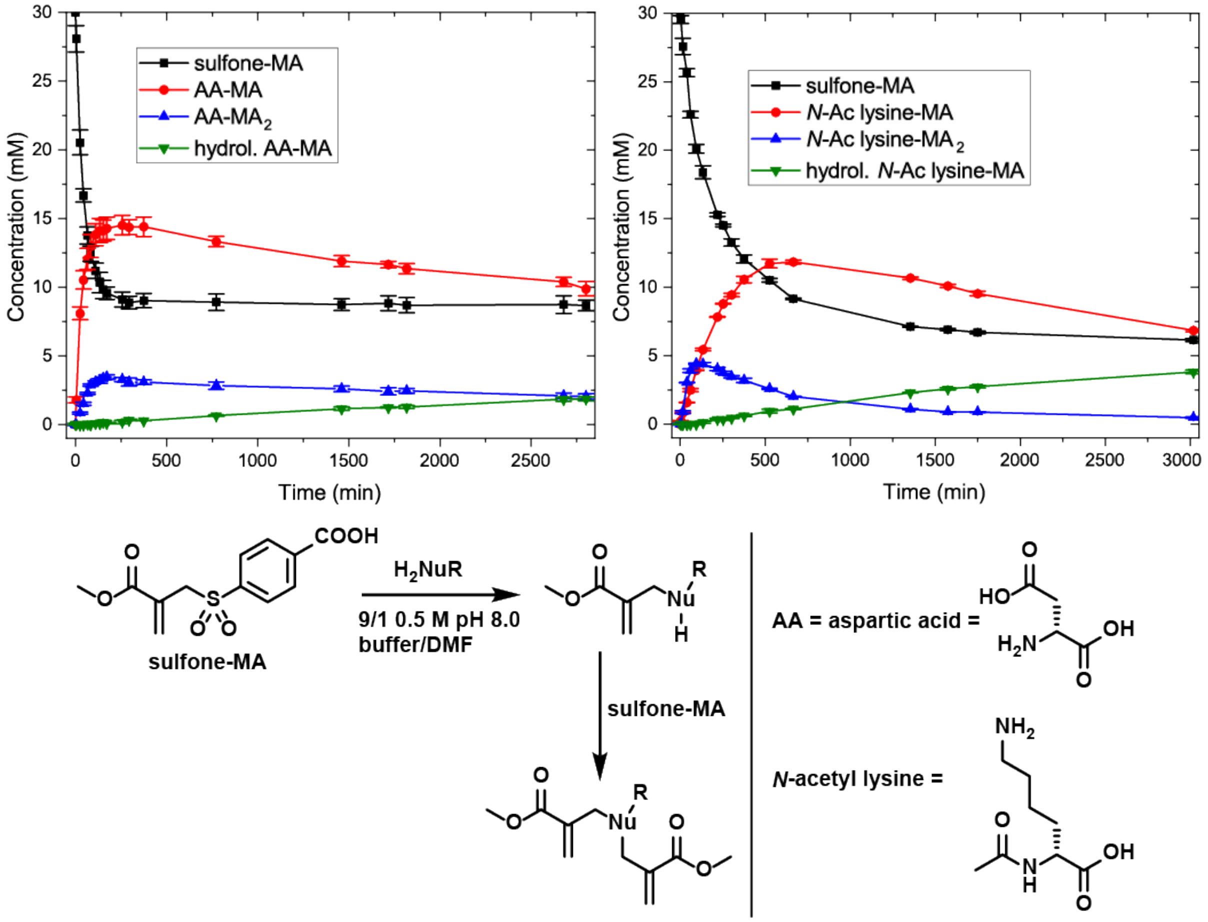

Figure S6: Reaction of sulfone-MA **4** with aspartic acid (left) and *N*-acetyl lysine (right). The graph shows the consumption of sulfone-MA **4** over time, along with the formation of the mono- and bis-adducts over time. The green line shows the hydrolysis of the mono-adducts over time.

Apart from comparing the kinetics to choose which nucleophile would best suit our system, we also looked at any potential side reactions. The main side reaction we found (besides the double addition of primary amines) was the hydrolysis of the substituted products (Figure S7). The comparatively slow hydrolysis of proline-MA was one of the points that made it a favorable candidate for our CRNs.

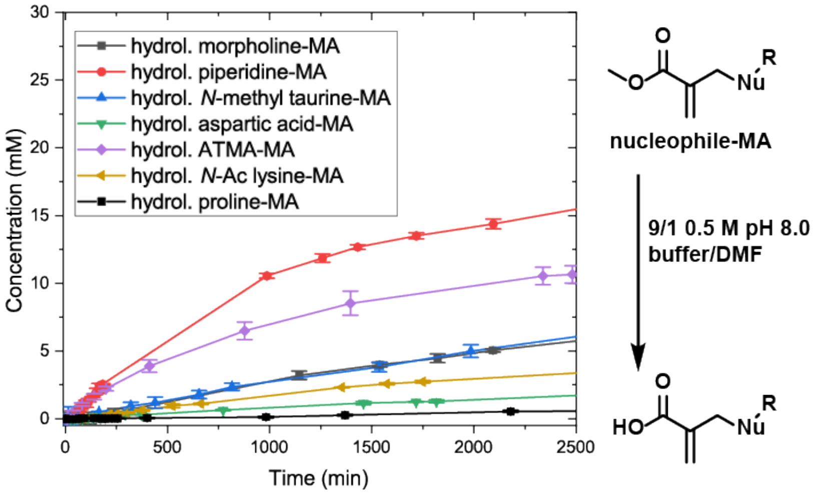

Figure S7: Hydrolysis of nucleophile-MAs (products of sulfone-MA **4** + nucleophile) over time. 3-amino benzoic acid is not shown, as no hydrolysis product was detected over this time scale.

Sulfone-MA 4 + additional proline: substitution reversibility and the fate of the sulfinate

As can be seen for the reaction of sulfone-MA **4** + nucleophiles (Figure S2 and Figure S6), different nucleophiles give different yields of nucleophile-MA, yet some sulfone-MA **4** always remains unreacted. We hypothesize that the substitution of sulfone-MA **4** under the release of the corresponding sulfinate salt is an equilibrium reaction. The sulfinate anion is nucleophilic enough to re-attack the nucleophile-MAs, displacing an amine. This would explain the lower yields with the less-nucleophilic amines (e.g., proline-MA **1** vs. morpholine-MA). To further support this hypothesis, upon the reaction of sulfone-MA **4** with 1 eq. proline, a further 5 eq. of proline were added to examine how the system behaves. We indeed found an increase in the yield of proline-MA **1**, suggesting the presence of an equilibrium between sulfone-MA **4** and nucleophile-MA. However, as demonstrated in Figure S8, an excess of nucleophiles should be avoided, as the increase in yield is negligible compared to the loss of active MA moieties due to the double addition of nucleophiles under the irreversible formation of double addition adducts.

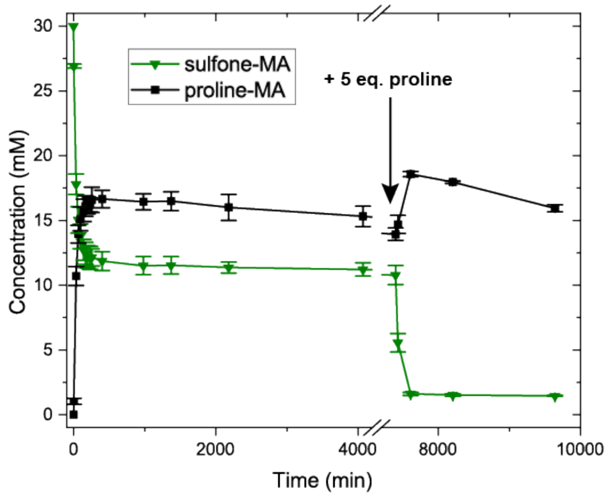

Figure S8: Reaction of sulfone-MA with 1.0 eq. proline and the addition of a further 5.0 eq proline.

Apart from these experiments, we managed to confirm the presence of the sulfinate of MBA via HRMS: found for $[M–H^+]^-$ 184.9907 (expected: 184.9914).

Furthermore, we found the sulfonate of MBA: found for $[M–H^+]^-$ 200.9858 (expected: 200.9863). It remains unclear whether the sulfonate formed during analysis in the LC-HRMS, or whether oxidation to sulfonate occurred before analysis due to the presence of atmospheric oxygen. In general, sulfinates (and even more so sulfinic acids) are prone to oxidation; however, aromatic sulfinates have been found to be somewhat more stable than their aliphatic counterparts.[12] In general, we assume that the sulfinate of MBA is relatively stable in solution. Otherwise, in the reaction of sulfone-MA **4** with nucleophiles, more nucleophile-MA would form over time as the released sulfinate degrades to yield sulfonate, which is not in equilibrium anymore.

Sulfone-MA 4 + thiol(s)

As expected, we found that not only amines can displace sulfinates by undergoing a substitution reaction with sulfone-MA **4**, but also thiols—as they are the stronger nucleophiles—giving sulfide-MA **2** and a sulfinate salt in the process (Scheme S2). This reaction is fast compared to the reaction with amines (i.e. full conversion within minutes). We chose an aromatic thiol (MBA, Figure S9) and an aliphatic thiol (3-mercapto propionic acid, Figure S10) to demonstrate this point.

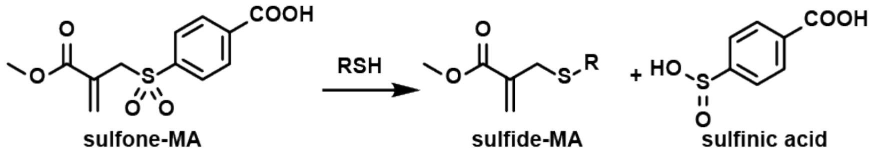

Scheme S2: Reaction of sulfone-MA with a thiol (RSH) to yield sulfide-MA and sulfinic acid (effectively sulfinate at pH 8).

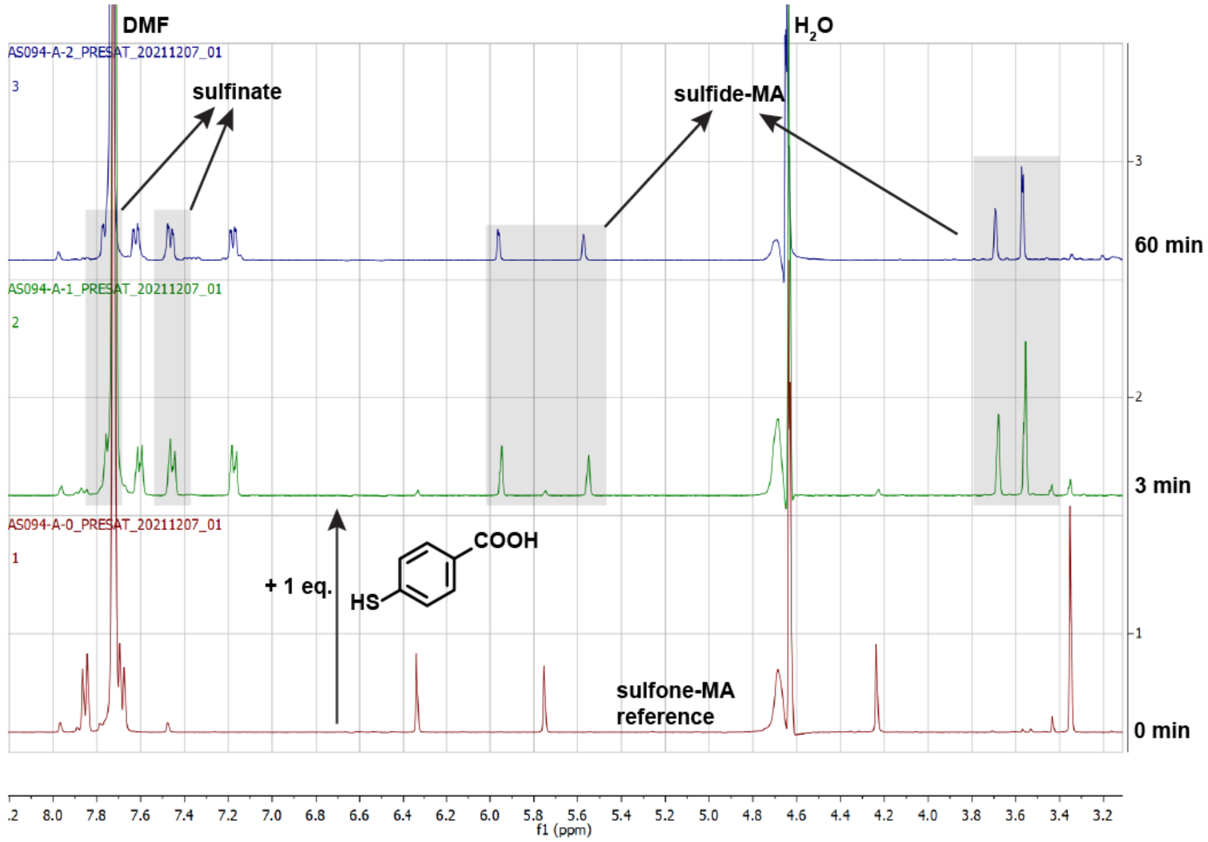

Figure S9: Reaction of sulfone-MA **4** with 1.0 eq. of MBA to yield sulfide-MA **2** and sulfinate. **Note:** The downfield aromatic sulfinate signal overlaps with the DMF signal.

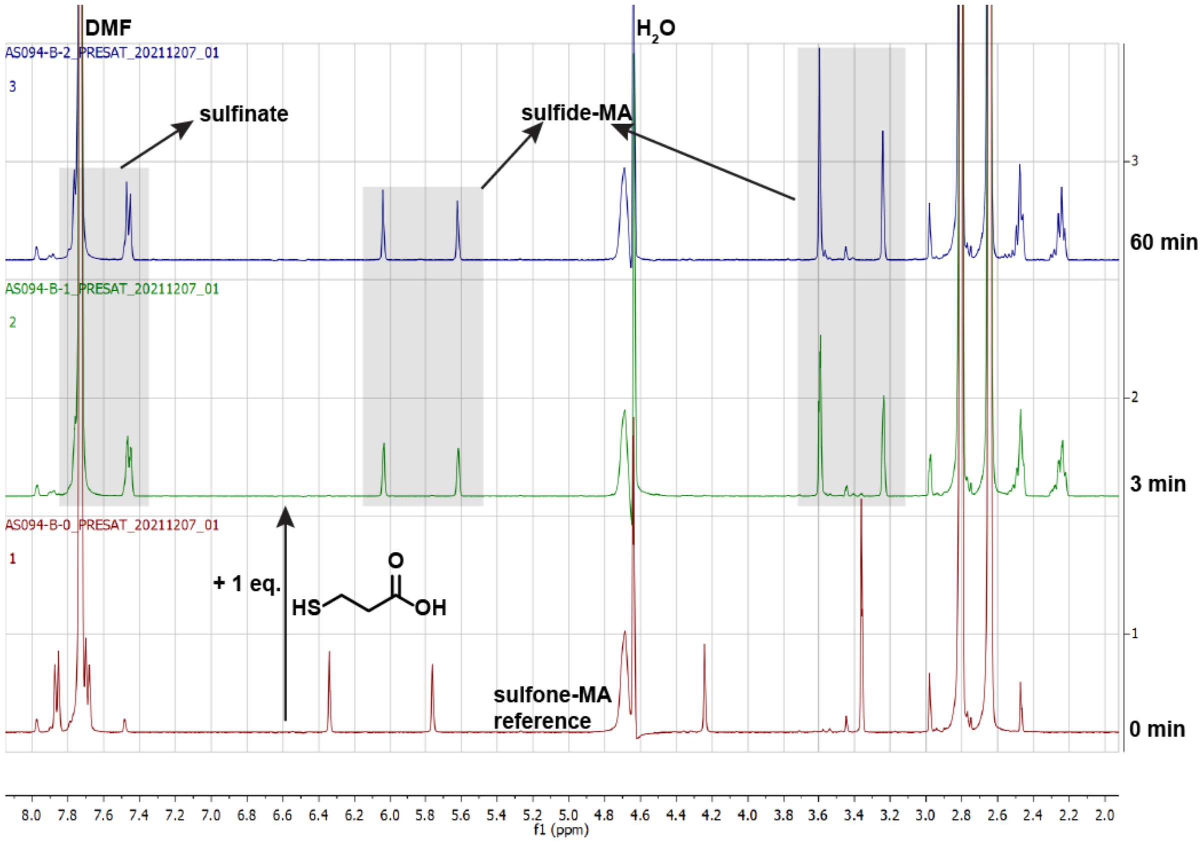

Figure S10: Reaction of sulfone-MA **4** with 1.0 eq. of 3-mercapto propionic acid to yield sulfide-MA **2** and sulfinate. **Note:** The downfield aromatic sulfinate signal overlaps with the DMF signal.

Sulfoxide-MA 3 + thiol

Analogously to the reaction between sulfone-MA **4** and thiols, the reaction between sulfoxide-MA **3** and thiols can also proceed, yielding a sulfide-MA **2** and a sulfenic acid which will further react according to Scheme S1 (see Figure S11). The same side products (i.e. sulfone-MA **4**, disulfide **5**) can also be found in the reaction of sulfoxide-MA **3** with proline (Figure 3d).

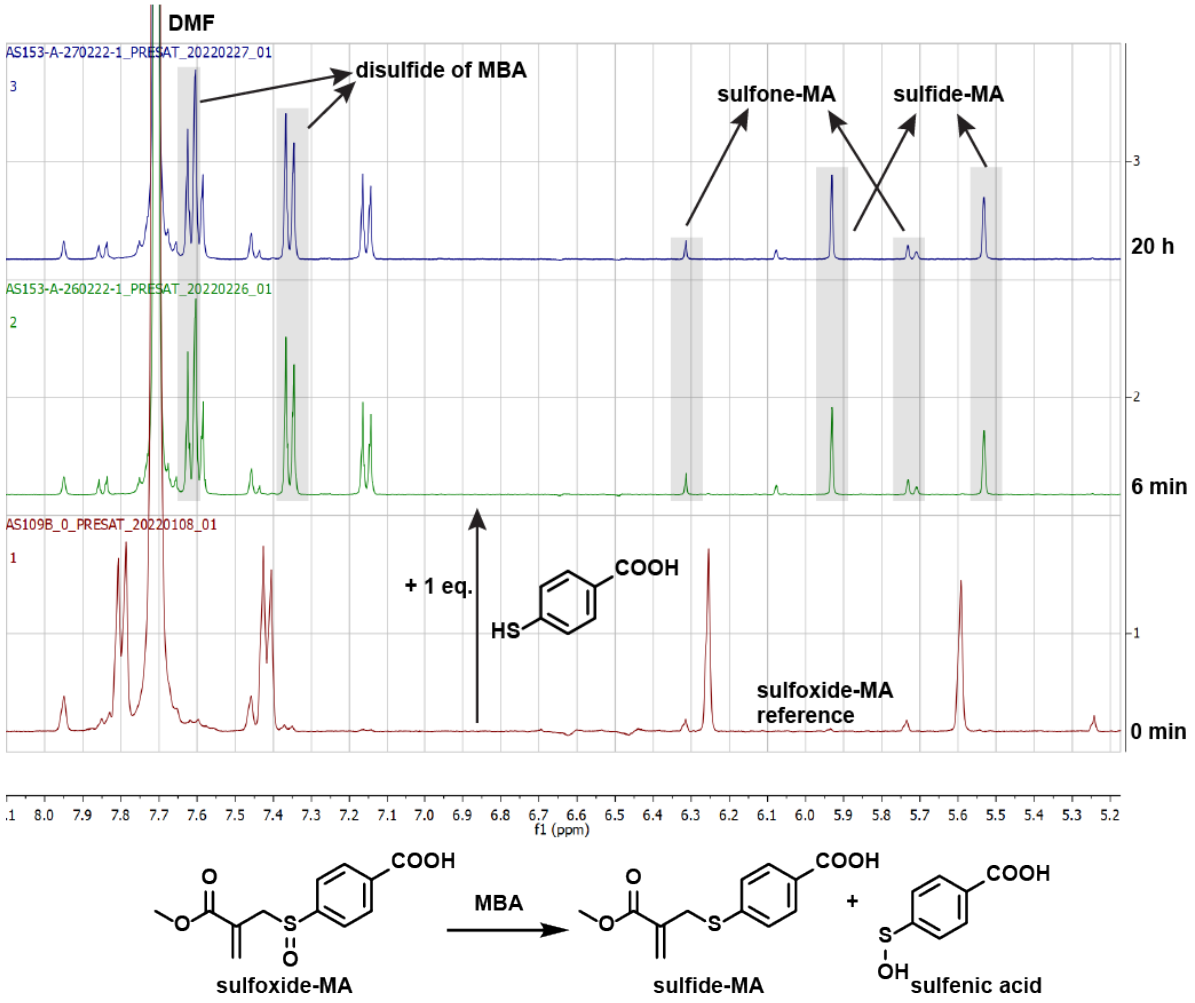

Figure S11: Reaction of sulfoxide-MA **3** with 1.0 eq. MBA to yield sulfide-MA **2**, along with the disulfide of MBA **5** (from the degradation of sulfenic acid) and small amounts of sulfone-MA **4** (from the degradation of sulfenic acid and further reaction with sulfoxide-MA **3**).

Sulfone network: simultaneous addition of MBA and Oxone

Unlike the stepwise addition experiments we tested in the main text (e.g. Figure 2d), we also decided to test a simultaneous addition of MBA and Oxone to see if the sulfone system can be run as a reaction cycle with strong oxidants. We found minimal recoveries of proline-MA **1** due to the swift reaction of Oxone with MBA to form the corresponding disulfide **5**. Furthermore, Oxone reacted both with proline and proline-MA **1** to form the nitrone and oxidized proline-MA, respectively (compare Scheme S1). This also explains why, even though proline-MA **1** can be recovered from oxidized proline-MA, the actual recoveries are minimal.

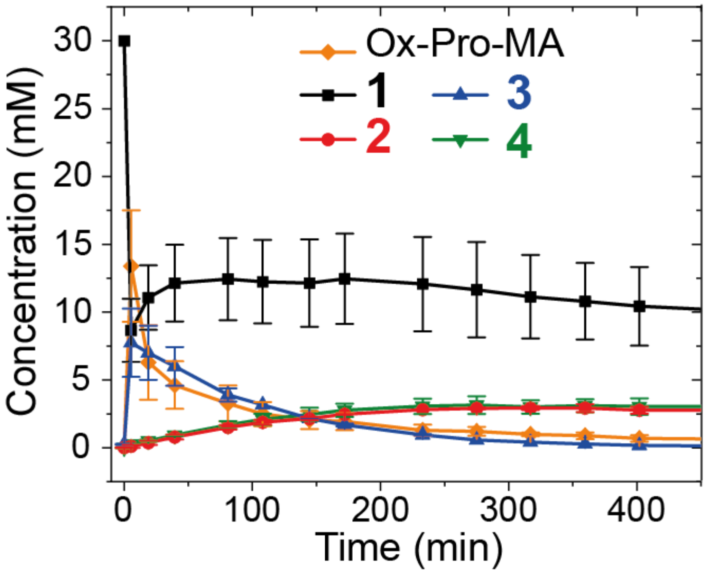

Figure S12: evolution of different species in the CRN upon simultaneous addition of MBA and Oxone to a solution of proline-MA **1**. The oxidation of thiol, proline, and proline-MA **1** outcompete the conjugate addition and elimination of thiol to proline-MA **1**.

Oxone: reference reactions

To demonstrate potential side reactions of our sulfone CRN, we performed several reference reactions of some species with Oxone. Here, we show how proline and proline-MA **1** react with Oxone. Furthermore, we show that acetate (side product of proline-MA **1** formation) and the used solvent mixture (i.e. buffer/DMF) are compatible with Oxone.

First, we subjected proline to 2.5 eq. Oxone. It has been shown in the literature that the oxidation of amino acids and subsequent decarboxylation to nitrones typically proceeds via *N*-hydroxylamino acids and a short-lived intermediate dihydroxylated species before decarboxylating.[13] It has also been shown that Oxone specifically is capable of producing a nitrone from proline.[3] In agreement with the literature data, we found the formation of the nitrone upon oxidation of proline. However, we were unable to achieve full conversion even with 2.5 eq. Oxone. Furthermore, we were unable to observe any potential intermediate species as the conversion occurred too fast to be tracked appropriately via ^{1}H-NMR (Figure S13).

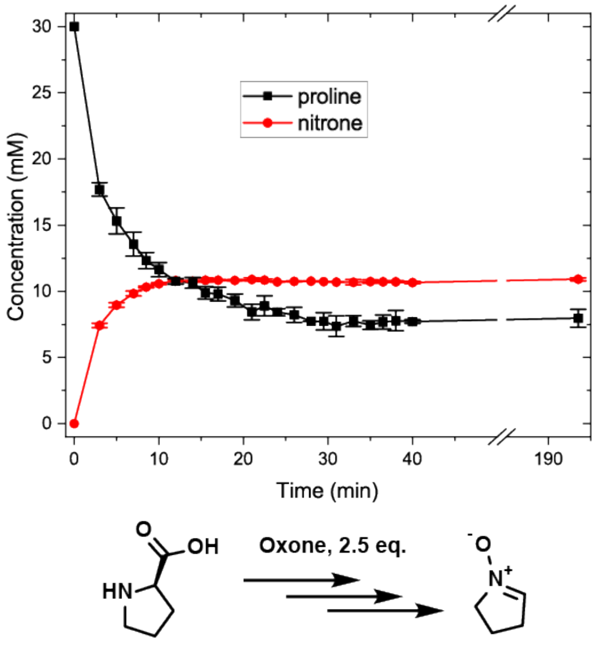

Figure S13: Reaction of proline with 2.5 eq. Oxone. The formation of nitrone was observed, tracked by the appearance of the characteristic double bond signal at ~ 7 ppm.

Besides the NMR results, we also managed to confirm the nitrone via HRMS from the reaction of proline-MA **1** with Oxone as a side product:

ESI-LC/HRMS (m/z): calculated for [$C_4H_8NO^+$] 86.0600, found: 86.0606.

Next, we subjected proline-MA **1** to Oxone. It can be seen that a swift oxidation of proline-MA **1** upon the addition of Oxone takes place to form Ox-Proline-MA (see Figure S14, see also Scheme S1). We also managed to confirm this oxidized species via HRMS:

ESI-LC/HRMS (m/z): calculated for [$C_{10}H_{16}NO_5^+$] 230.1023, found: 230.1015.

In alignment with the mechanism proposed in literature,[13] a decarboxylation, as in the oxidation of free proline, cannot take place due to the tertiary nature of the amine in the proline-MA **1** species. Therefore, the oxidation stops at the stage of the quaternary oxidized proline-MA.

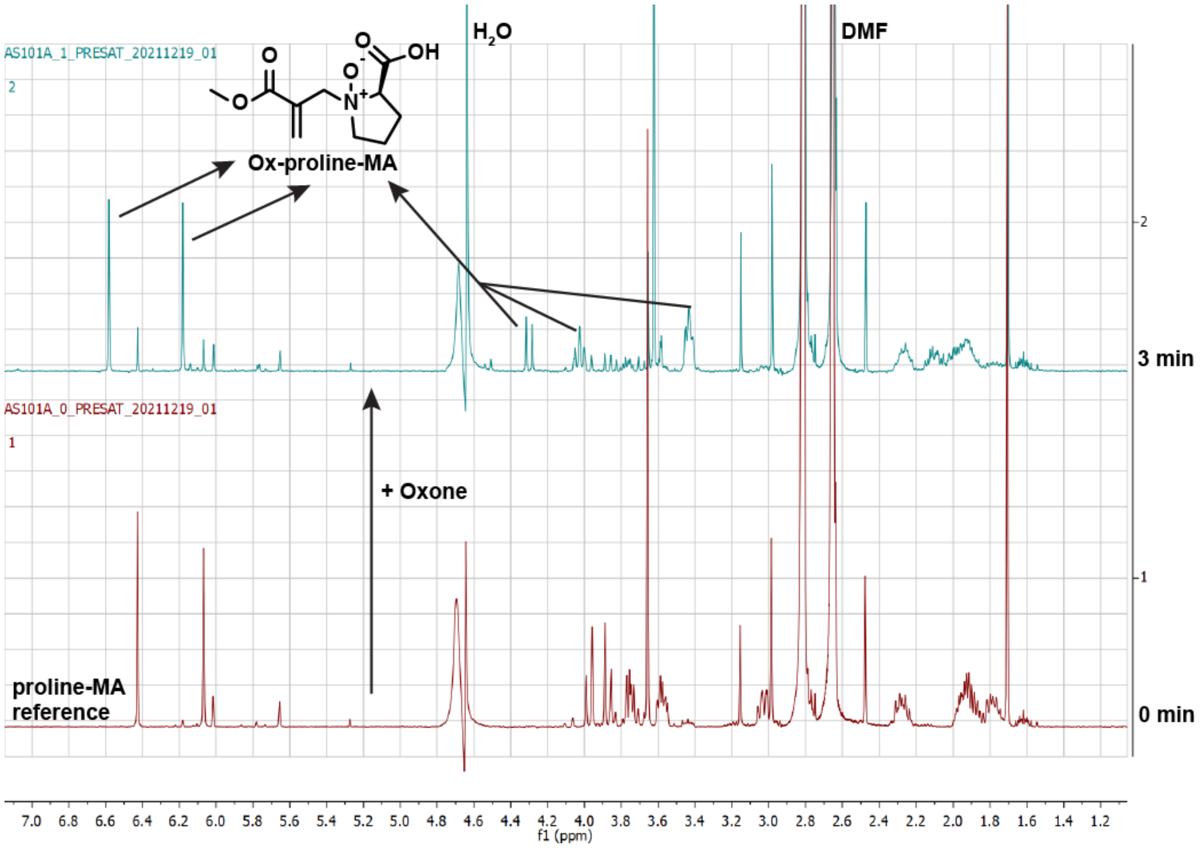

Figure S14: Reaction of proline-MA **1** with Oxone to yield oxidized proline-MA.

Lastly, we also subjected sodium acetate, as well as our solvent mixture (9/1 0.5 M pH 8.0 potassium phosphate buffer/DMF), to Oxone. Neither of those experiments showed any changes over time, ruling out any potential cross-reactivity between acetate (side product of proline-MA **1** preparation) and Oxone, as well as the used solvent and Oxone.

Recovery of Proline-MA 1 from Ox-Proline-MA

As shown in Scheme S1, we have added a backward arrow leading back from oxidized Proline-MA to proline-MA **1**. We have found that upon oxidation of proline-MA **1**, the oxidized species becomes electron-deficient, and hence proline can re-attack, leading to the recovery of proline-MA **1**. As can be seen in Figure S15, proline-MA **1** quickly gets oxidized to form oxidized proline-MA (compare Scheme S1). Further, this signal decreases in intensity, and a new signal appears. We hypothesize that the oxidized proline-MA undergoes isomerization via nucleophilic attack of the amine-*N*-oxide on a second oxidized proline-MA to yield the *O*-bonded form as the thermodynamic product.

Upon addition of proline, proline-MA **1** gets recovered quickly from the oxidized proline-MA species via nucleophilic attack and substitution of *N*-hydroxyproline. The reason why this recovery is less efficient when trying to run the Oxone CRN as a reaction cycle (Figure S12) is the fact that proline, which is needed for the recovery of proline-MA **1**, also gets consumed by the Oxone that is added to the reaction mixture.

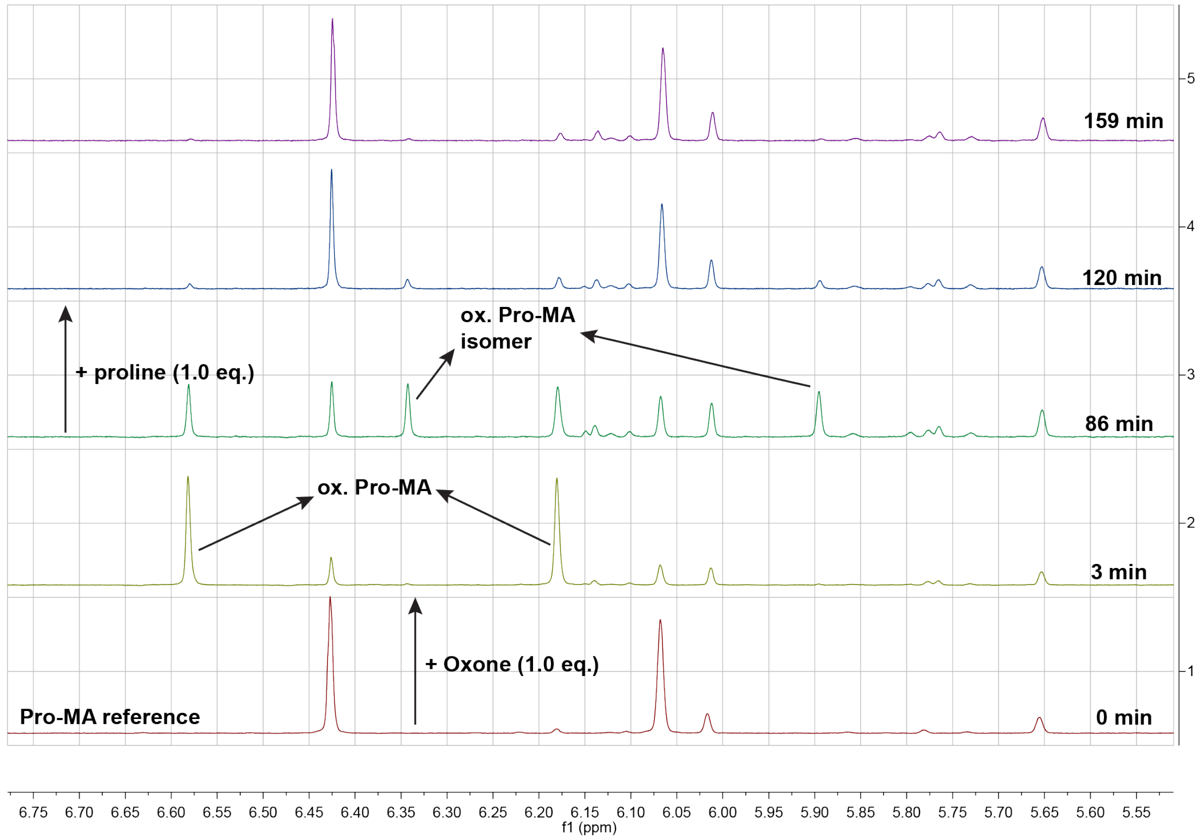

Figure S15: Oxidation of Proline-MA **1** with Oxone and subsequent addition of proline to recover Proline-MA **1**.

Stepwise addition: comparison of oxidants

To directly compare the efficiency of different oxidants of recovering proline-MA **1** over time, we plotted the recoveries of proline-MA **1** over time from stepwise addition experiments, adding 2.5 eq. of oxidant in each case. In each case, we started from the usual 30 mM proline-MA **1** stock solution and added 1.0 eq. MBA, followed by adding a certain amount of oxidant (specified below).

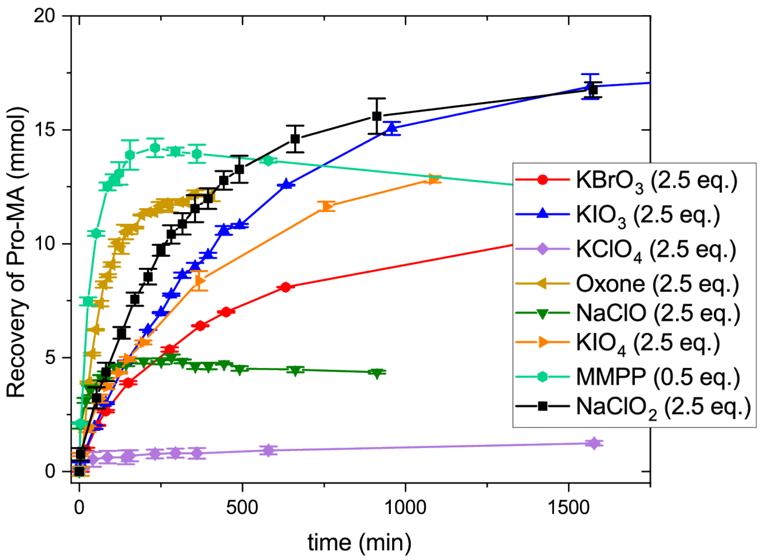

Figure S16: Comparison of different oxidants in the recovery of proline-MA **1** upon addition of 1.0 eq. of MBA to a 30 mM proline-MA stock solution, followed by the addition of the oxidant.

It can clearly be seen that the rates of recovery, the maximum level of recovery, and the product stability over time all vary widely, depending on the oxidant. $KClO_4$ (violet line with diamonds) and $KClO_3$ (omitted for clarity) do not work in recovering proline-MA **1** from sulfide-MA **2**, likely due to their weak oxidation strength under the used conditions compared to the other oxidants used. $KBrO_3$ and KIO_3 recover proline-MA **1** relatively slowly, as the recovery mechanism proceeds purely via oxidation of free thiol from equilibrium. As the concentration of free thiol and the rate of equilibration limit the overall rate of recovery, precise control of the recovery kinetics via variation of the oxidant quantity is not possible in the sulfide pathway. The oxidants that can oxidize sulfide-MA **2** to sulfoxide-MA **3** and/or sulfone-MA **4** lead to a more rapid recovery of proline-MA **1**, as the recovery of **1** depends on the direct displacement of sulfenic and/or sulfinic acid by proline instead of release of MBA. As we have shown, proline-MA **1** can be recovered similarly fast from sulfoxide-MA **3** and sulfone-MA **4**.

As the formation of sulfoxide-MA **3** is faster than that of sulfone-MA **4** with strong oxidants, the amount of oxidant used is another crucial factor. A good example is MMPP. While adding 2.5 eq. (omitted for clarity) led to a whole range of side reactions, as well as low and slow recovery rates, the use of 0.5 eq. led to a fast recovery of proline-MA **1**. However, the stability of recovered proline-MA was low over time. Likewise, for sodium hypochlorite, we also observed side reactivity, low recovery rates, and low stability of recovered proline-MA **1** over time. Oxone, on the other hand, led to a relatively fast recovery rate, high recovery level, and improved stability over time.

Hence, it can be concluded that by varying the applied oxidant, one has control over which pathway the recovery will take (i.e. slow sulfide pathway vs the much faster sulfoxide and sulfone pathways). Furthermore, the amount of strong oxidant (i.e. sulfone pathway) used can kinetically bias the pathway; using stoichiometric or substoichiometric amounts of oxidant can bias recovery mode more towards the sulfoxide pathway while using an excess of oxidant will bias the recovery mode more towards the sulfone pathway. These factors strongly influence side products, recovery rates, and the kinetics of the recovery.

Sulfoxide-MA stability study

Unlike sulfide-MA **2** and sulfone-MA **4**, sulfoxide-MA **3** was neither stable in its pure form nor in solution for prolonged periods of time. We found a comparatively swift degradation of sulfoxide-MA **3** in solution (9/1 buffer/DMF), likely either via a solvent-induced addition-substitution SN2' reaction or a two-step process in which a [2,3]-sigmatropic rearrangement to yield a sulfenate ester is followed by hydrolysis. Both of these mechanisms yield the same products. Accordingly, we observed HO-MA as the main side product, along with the common sulfenic acid degradation products mentioned previously (i.e. sulfide-MA **2**, sulfone-MA **4**, disulfide of MBA **5**). Furthermore, this degradation (Figure S17) also explains why oxidants that should selectively proceed via the sulfoxide pathway (main text, Figure 1, center) also show the formation of minor amounts of sulfone-MA **4** over time—not due to over-oxidation but due to sulfoxide-MA degradation instead. This sulfone-MA **4** formation may, however, be somewhat suppressed in the presence of oxidants compared to the free degradation of sulfoxide-MA **3**, as oxidants can react with the sulfenic acid, reducing the formation of its degradation products that would otherwise form in a non-oxidizing environment such as in this experiment (Figure S17).

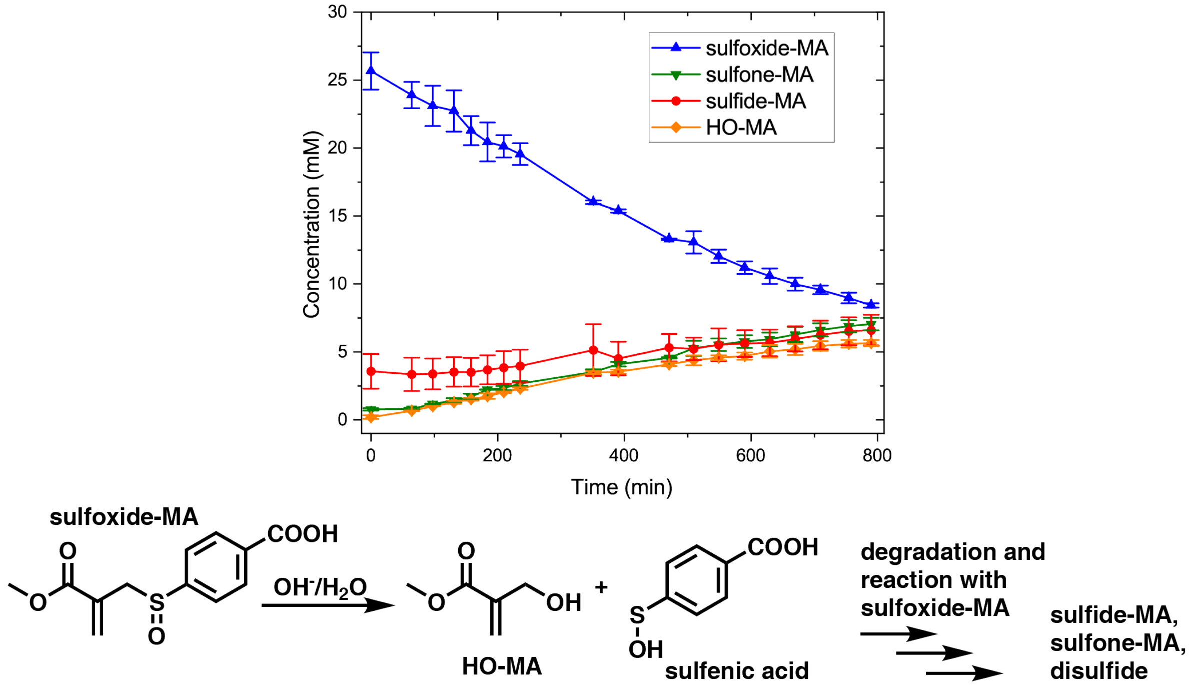

Figure S17: Degradation of sulfoxide-MA **3** in 9/1 0.5 M pH 8.0 buffer/DMF. Unlike sulfide-MA **2** and sulfone-MA **4**, sulfoxide-MA **3** is unstable in solution and degrades to yield HO-MA and the common side products of sulfenic acid degradation (refer to Scheme S1). **Note:** The initial presence of sulfide-MA **2** is due to incomplete oxidation of this batch of sulfoxide-MA **3**. However, this likely plays no role in the degradation of sulfoxide-MA **3**.

Bromate: reference reactions

To further corroborate the way in which the third CRN proceeds (Figure 1, right scheme, main text), we reacted potassium bromate with sulfide-MA, with MBA, and with proline. We found that, unlike stronger oxidants, bromate is incapable of oxidizing sulfide-MA, neither to

sulfoxide-MA nor sulfone-MA (see Figure S18). This was expected, as bromate is typically not the active oxidizer but requires acidic conditions and the presence of bromide ions to comproportionate into the more reactive bromine. At pH 8 and without bromide ions, these conditions are not given. Also, unlike Oxone, bromate does not react with proline (see Figure S19), avoiding the side reaction of forming oxidized proline-MA as it is found with stronger oxidants. However, bromate efficiently oxidizes the thiol MBA to form the corresponding disulfide of MBA **5** (see Figure S20).

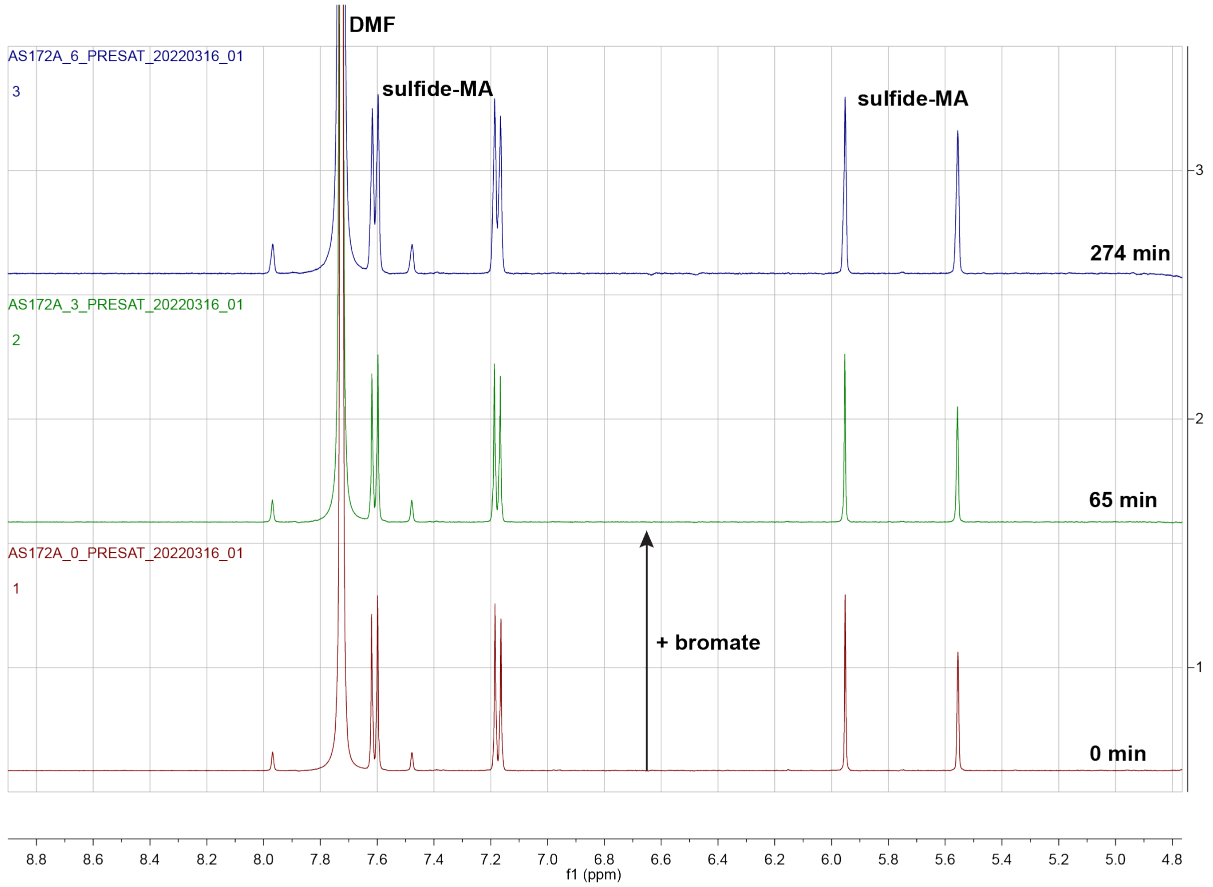

Figure S18: sulfide-MA **2** with 2.5 eq. potassium bromate. No oxidation of sulfide-MA **2** could be observed at pH 8.0 over the observed timeframe.

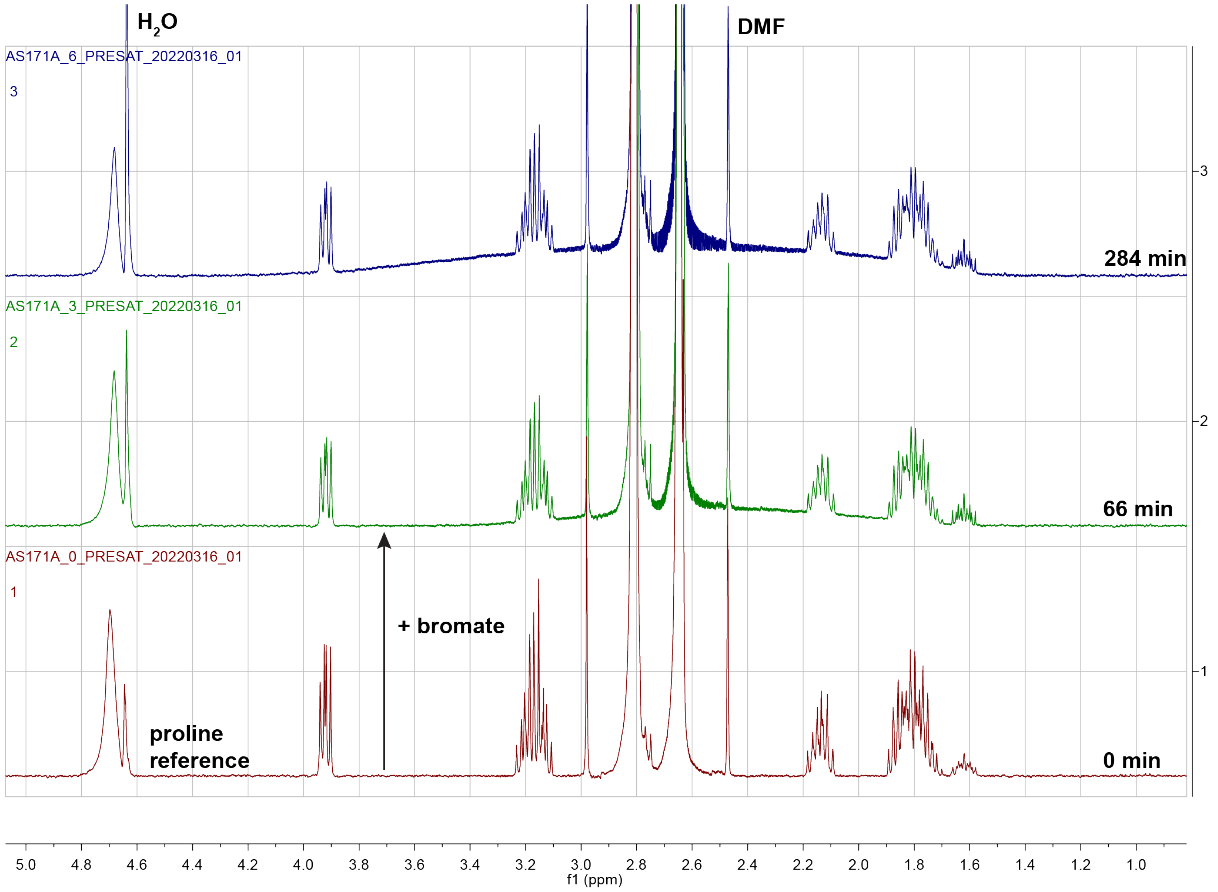

Figure S19: proline with 2.5 eq. potassium bromate. No oxidation of proline could be observed at pH 8.0 over the observed timeframe.

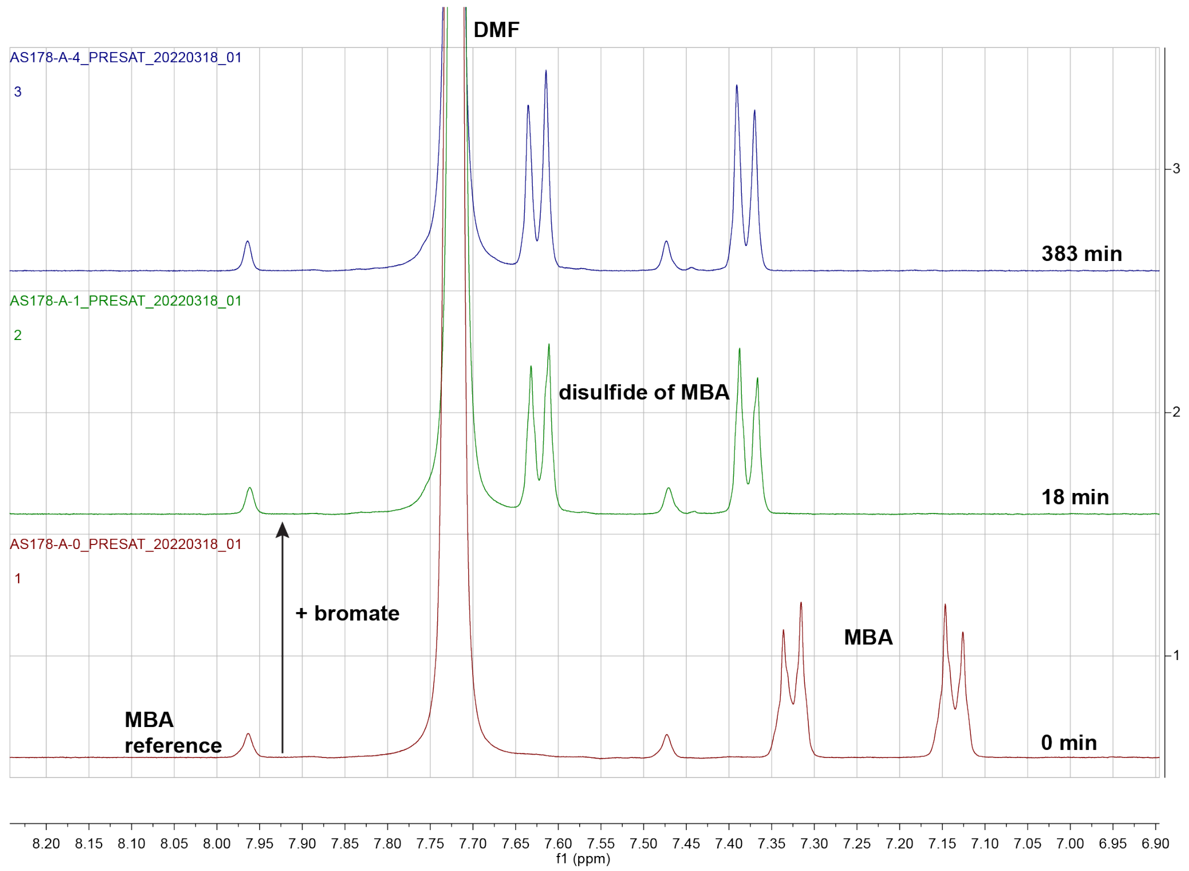

Figure S20: MBA with 2.5 eq. potassium bromate. MBA was quickly oxidized to the corresponding disulfide of MBA **5**.

Addition reactions without substitution: Loss of double bond functionality

As can be seen in Scheme S1, one of the side reactions which causes a loss of double bond functionality is the addition of a nucleophile (amine, thiol) to any MA-species without a substitution (i.e. expelling of leaving group) taking place. This side reaction is difficult to track via NMR due to the absence of acrylic signals and the strong overlap of the remaining signals. Hence, we resorted to HRMS to prove the formation of double adducts.

First, we investigated to the behavior of Proline-MA **1** over time in the presence of 1.0 eq. free proline (Figure S21). We found a decrease of roughly 15 % in total MA-moiety concentration over 29 h when adding 1.0 eq. proline to a 30 mM solution of Proline-MA **1**. Quantifying the double adduct via NMR is challenging due to the strong overlap and absence of acrylic signals. However, we managed to identify the double adduct via HRMS as the main side product:

ESI-LC/HRMS (m/z): calculated for $[C_{15}H_{24}N_2O_6]^+$ 329.1707, found: 329.1693.

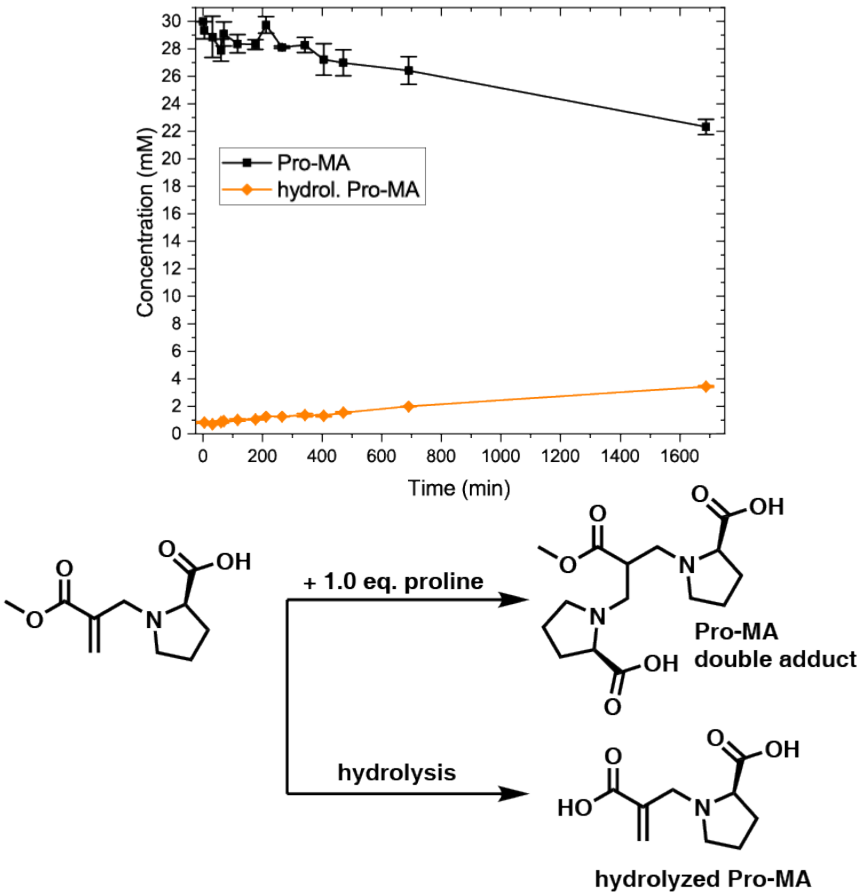

Figure S21: Double addition of proline to proline-MA **1**, along with background hydrolysis. The overall concentration of double bond moieties (i.e. proline-MA **1** + hydrol. Proline-MA) decreases from 30.0 mM to 25.8 mM.

Next, we tested the behavior of sulfoxide-MA **3** and sulfone-MA **4** in the presence of 2.0 eq. free proline. As the reaction of **3** with proline will also generate sulfide-MA **2** in the process, these experiments also serve to probe for the double addition to **2**. This also explains the non-quantitative yield of proline-MA **1** + MBA in stepwise additions; a part (usually ~ 10 %) gets lost due to the addition side reaction. Indeed, we managed to confirm all species proposed in Scheme S1 via HRMS:

- Addition of proline to sulfoxide-MA **3**:
  ESI-LC/HRMS (m/z): calculated for $[C_{17}H_{22}NO_7S]^+$ 384.1111, found: 384.1153.

- Addition of proline to sulfone-MA **4**:
  ESI-LC/HRMS (m/z): calculated for $[C_{17}H_{22}NO_8S]^+$ 400.1061, found: 400.1046; calculated for $[C_{17}H_{20}NO_8S]^-$ 398.0915, found: 398.0918.

- Addition of 4-mercapto benzoic acid (MBA) to sulfide-MA **2**:
  ESI-LC/HRMS (m/z): calculated for $[C_{19}H_{17}O_6S_2]^-$ 405.0472, found: 405.0475.

- Addition of MBA to sulfoxide-MA **3**:
  ESI-LC/HRMS (m/z): calculated for $[C_{19}H_{19}O_7S_2]^+$ 423.0567, found: 423.0550; calculated for $[C_{19}H_{17}O_7S_2]^-$ 421.0421, found: 421.0425.

- Addition of MBA to sulfone-MA **4**:
  ESI-LC/HRMS (m/z): calculated for $[C_{19}H_{19}O_8S_2]^+$ 439.0516, found: 439.0502; calculated for $[C_{19}H_{17}O_8S_2]^-$ 437.0370, found: 437.0373.

Phosphine + MA

One of the most dominant side reactions we found in the flow experiments with TCEP was the addition of proline-MA **1** to form a TCEP-MA adduct. To confirm this, we measured a reference ^{31}P spectrum of TCEP and added 1.0 eq. Br-MA. We found the same signals as in the flow experiments, both in ^{1}H and ^{31}P spectra. Interestingly, two phosphorus signals appeared upon the addition of Br-MA, with close chemical shifts. We hypothesize that these are the mono- and double-adduct of TCEP to Br-MA. Other reagents, such as borohydrides, may be an alternative recovery reagent in batch conditions, however, preparing stock solutions for flow experiments is difficult due to their *in situ* degradation.[14,15]

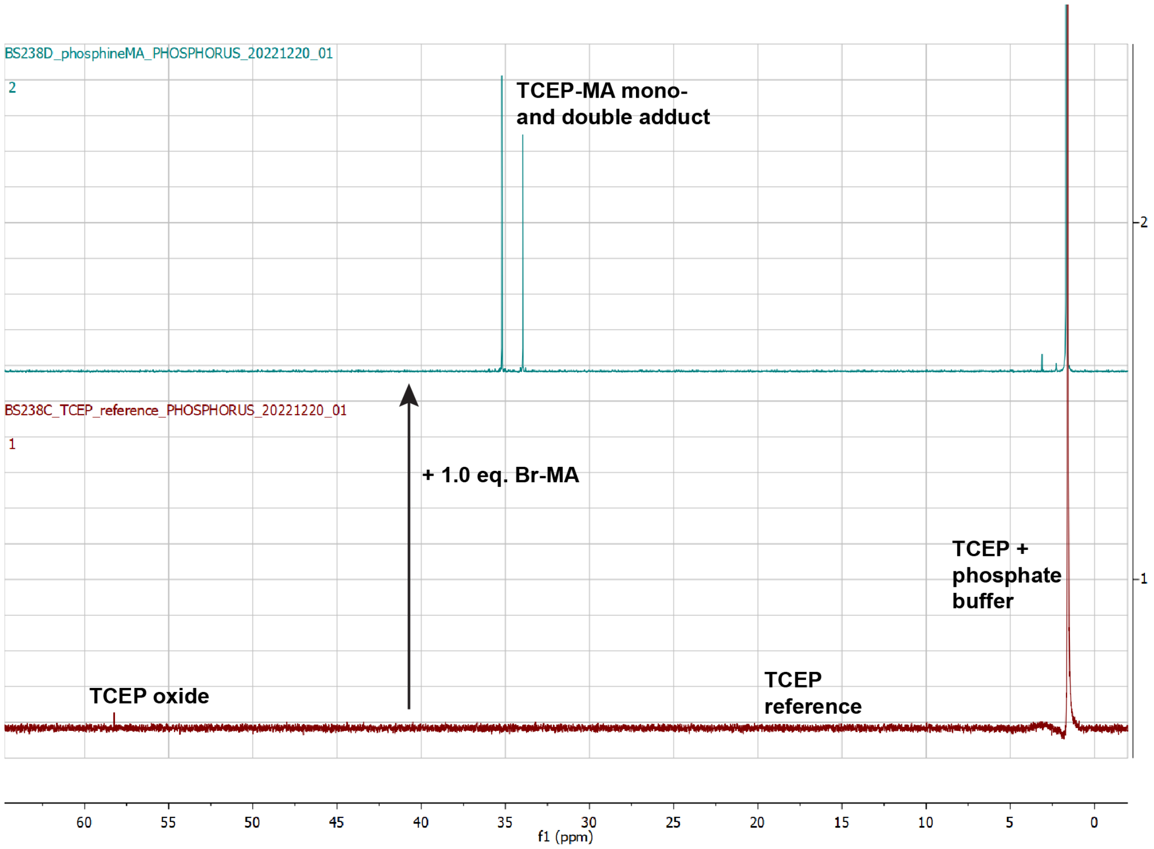

Figure S22: Addition of 1.0 eq. Br-MA to TCEP, yielding two new signals of TCEP-MA adducts, likely the mono- and bis-adducts.

Bromate RC with aliphatic thiol

After we managed to identify satisfying conditions for our bromate RC with our typically employed thiol MBA and proline, we tested the influence of using a different thiol. We started the cycle with proline but using an aliphatic thiol—3-mercaptopropionic acid—instead of MBA.

We found that with 3-mercaptopropionic acid (3MPrA), unlike with MBA, no significant recovery could be observed (see Figure S23). We hypothesize that this has to do with the acidity of the employed thiol. Under nucleophilic attack from proline, MBA is a much better leaving group due to the high charge stabilization of its thiolate. The much lower acidity probably pushes the equilibrium between proline-MA **1** and 3MPrA-MA even further toward the side of the sulfide, strongly disfavoring the thiolate as a leaving group and hence not leading to any recovery of initial proline-MA **1** over the observed timescale.

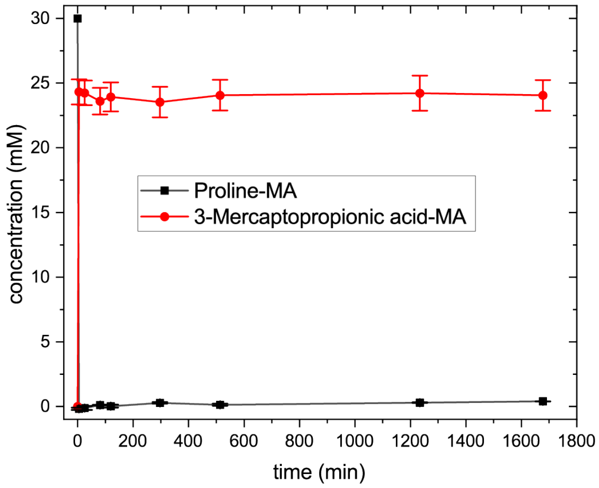

Figure S23: simultaneous addition of 1.0 eq. of 3MPrA and 2.5 eq. potassium bromate to a 30 mM solution of proline-MA **1**. While the sulfide-MA **2** forms efficiently and fast, as with MBA, no recovery of proline-MA **1** can be observed over time.

References

(1) Koo, B. S.; Lee, C. K.; Lee, K. J. Oxidation of Benzyl Alcohols with Oxone® and Sodium Bromide. *Synth. Commun.* **2002**, *32* (14), 2115–2123. https://doi.org/10.1081/SCC-120005418.

(2) Murahashi, S.-I.; Shiota, T. Short-Step Synthesis of Amino Acids And N-Hydroxyamino Acids From Amines. *Tetrahedron Lett.* **1987**, *28* (44), 5241–5244.

(3) Gella, C.; Ferrer, È.; Alibés, R.; Busqué, F.; De March, P.; Figueredo, M.; Font, J. A Metal-Free General Procedure for Oxidation of Secondary Amines to Nitrones. *J. Org. Chem.* **2009**, *74* (16), 6365–6367. https://doi.org/10.1021/jo901108u.

(4) Ligon, S. C.; Seidler, K.; Gorsche, C.; Griesser, M.; Moszner, N.; Liska, R. Allyl Sulfides and α-Substituted Acrylates as Addition-Fragmentation Chain Transfer Agents for Methacrylate Polymer Networks. *J. Polym. Sci. Part A Polym. Chem.* **2016**, *54* (3), 394–406. https://doi.org/10.1002/pola.27788.

(5) Denmark, S. E.; Forbes, D. C.; Hays, D. S.; DePue, J. S.; Wilde, R. G. Catalytic Epoxidation of Alkenes with Oxone. *J. Org. Chem.* **1995**, *60*, 1391–1407. https://doi.org/10.1021/jo971781y.

(6) Nagy, P.; Winterbourn, C. C. Redox Chemistry of Biological Thiols. *Adv. Mol. Toxicol.* **2010**, *4* (C), 183–222. https://doi.org/10.1016/S1872-0854(10)04006-3.

(7) Kice, J. L.; Venier, C. G.; Large, G. B.; Heasley, L. Mechanisms of Reactions of Thiolsulfinates (Sulfenic Anhydrides). III. The Sulfide-Catalyzed Disproportionation of Aryl Thiosulfinates. *J. Am. Chem. Soc.* **1969**, *91* (8), 2028–2035. https://doi.org/10.1021/jo01269a051.

(8) Matos, M. J.; Oliveira, B. L.; Martínez-Sáez, N.; Guerreiro, A.; Cal, P. M. S. D.; Bertoldo, J.; Maneiro, M.; Perkins, E.; Howard, J.; Deery, M. J.; Chalker, J. M.; Corzana, F.; Jiménez-Osés, G.; Bernardes, G. J. L. Chemo- and Regioselective Lysine Modification on Native Proteins. *J. Am. Chem. Soc.* **2018**, *140* (11), 4004–4017. https://doi.org/10.1021/jacs.7b12874.

(9) White, D. H.; Noble, A.; Booker-Milburn, K. I.; Aggarwal, V. K. Diastereoselective Photoredox-Catalyzed [3 + 2] Cycloadditions of N-Sulfonyl Cyclopropylamines with Electron-Deficient Olefins. *Org. Lett.* **2021**, *23* (8), 3038–3042. https://doi.org/10.1021/ACS.ORGLETT.1C00711/ASSET/IMAGES/LARGE/OL1C00711_0005.JPEG.

(10) Spiro, M. The Standard Potential of the Peroxosulphate/Sulphate Couple. *Electrochim. Acta* **1979**, *24* (3), 313–314. https://doi.org/10.1016/0013-4686(79)85051-3.

(11) Challenger, S.; Derrick, A.; Mason, C. P.; Silk, T. V. Stereoselective Synthesis of a Candoxatril Intermediate via Asymmetric Hydrogenation. *Tetrahedron Lett.* **1999**, *40*, 2187–2190.

(12) Truce, W. E.; Roberts, F. E. A Convenient Synthesis of Aromatic and Aliphatic Sodium Sulfinates. *J. Org. Chem.* **1963**, *28* (2), 593–594. https://doi.org/10.1021/jo01037a524.

(13) Murahashi, S.-I.; Imada, Y.; Ohtake, H. Tungstate-Catalyzed Decarboxylative Oxidation of N-Alkyl-Alpha-Amino Acids: An Efficient Method for Regioselective Synthesis of Nitrones. *J. Org. Chem.* **1994**, *59*, 6170–6172.

(14) Stahl, C. R.; Siggia, S. Determination of Organic Disulfides by Reduction with Sodium Borohydride. *Anal. Chem.* **1957**, *29* (1), 154–155. https://doi.org/10.1021/AC60121A046/ASSET/AC60121A046.FP.PNG_V03.

(15) Duane Brown, W. Reduction of Protein Disulfide Bonds by Sodium Borohydride. *Biochim. Biophys. Acta* **1960**, *44* (C), 365–367. https://doi.org/10.1016/0006-3002(60)91579-1.

Appendix

^{1}H-NMR spectrum of methyl (2-acetoxymethyl)acrylate (AcO-MA) in $CDCl_3$:

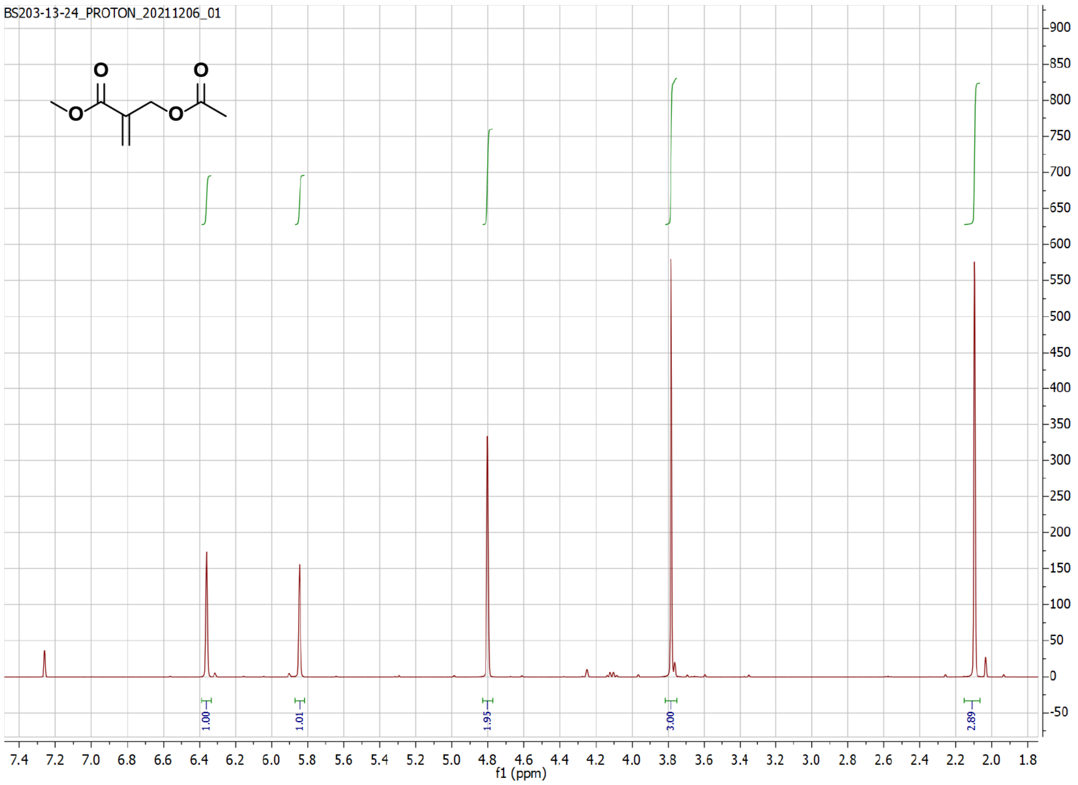

^{1}H-NMR, COSY, ^{13}C, HMBC and HSQC spectra of (2-(methoxycarbonyl)allyl)-L-proline (Proline-MA) in MeOD-d_4:

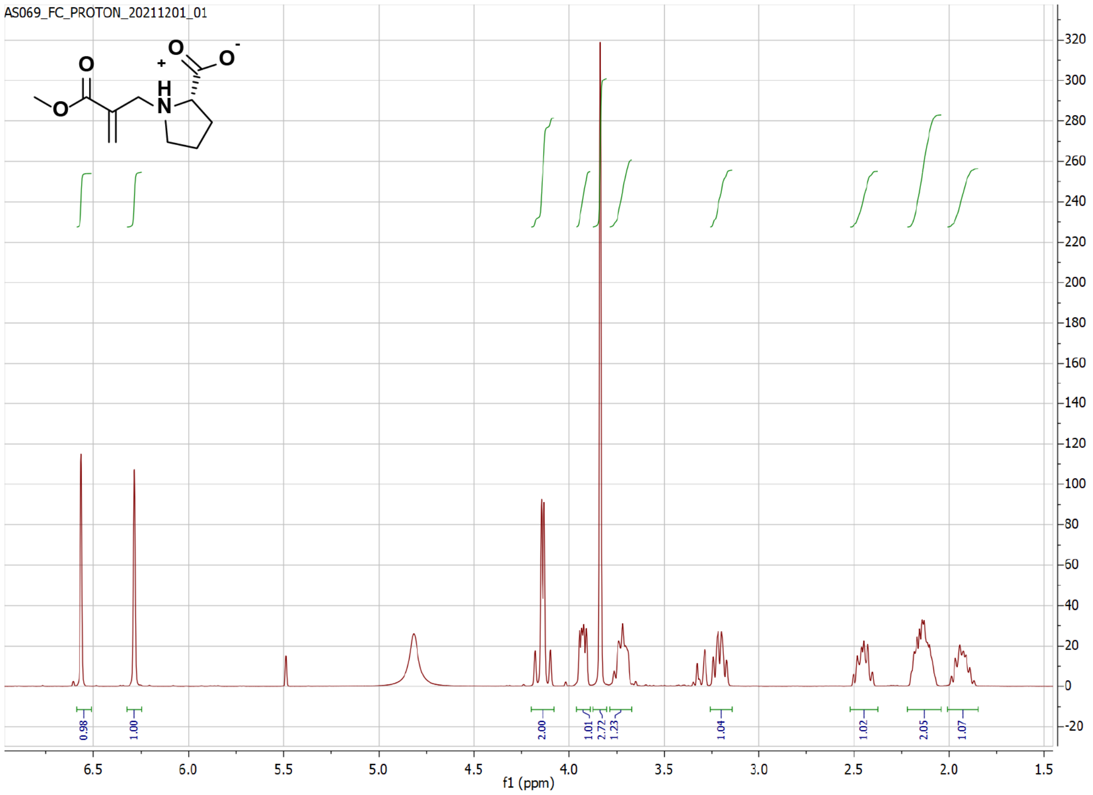

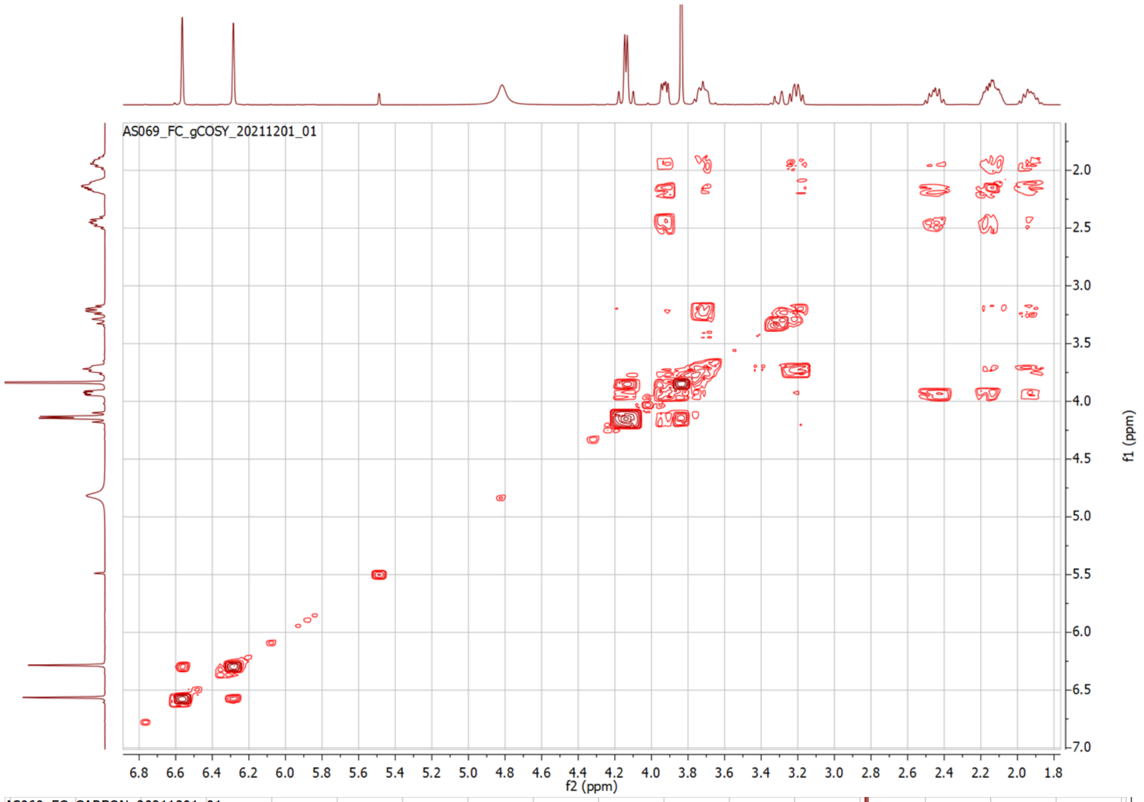
AS069_FC_gCOSY_20211201_01
f2 (ppm)
f1 (ppm)

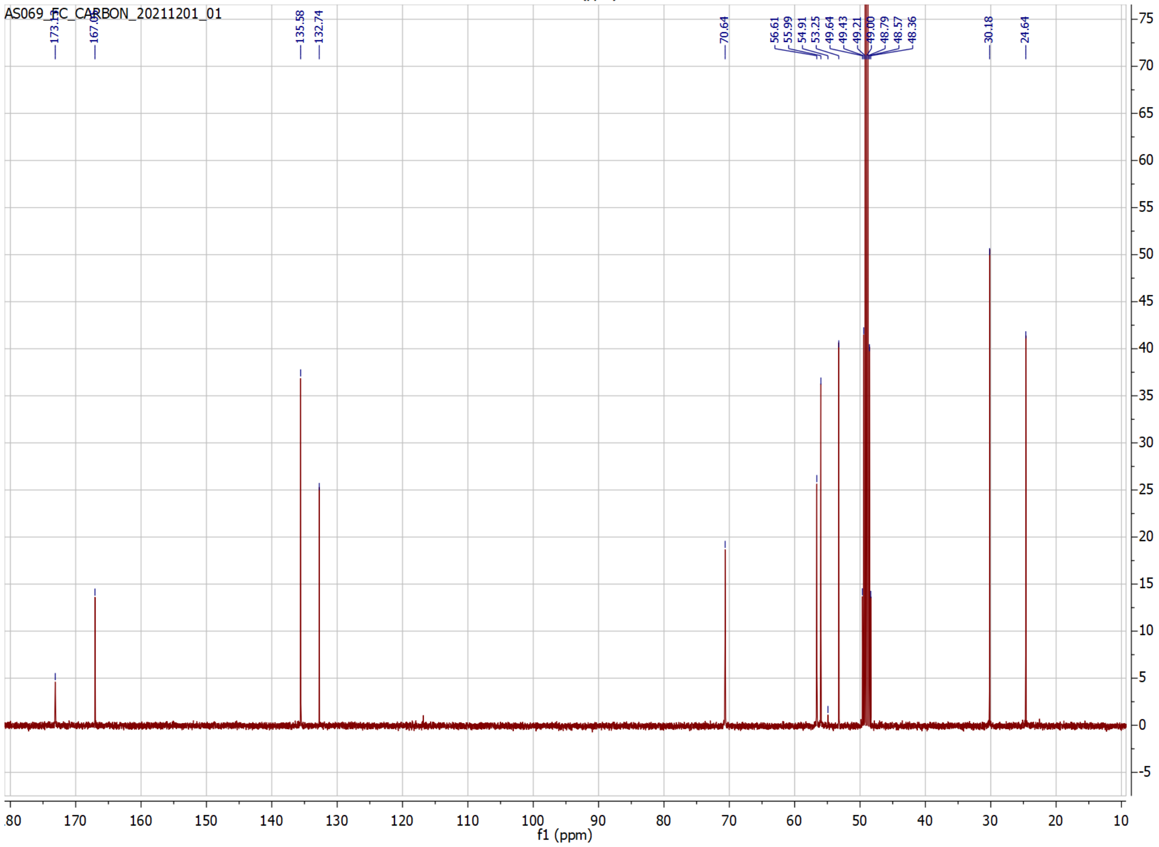
AS069_FC_CARBON_20211201_01
173.1
167.0
135.58
132.74
70.64
56.61
55.99
54.91
53.25
49.64
49.43
49.21
49.00
48.79
48.57
48.36
30.18
24.64
f1 (ppm)

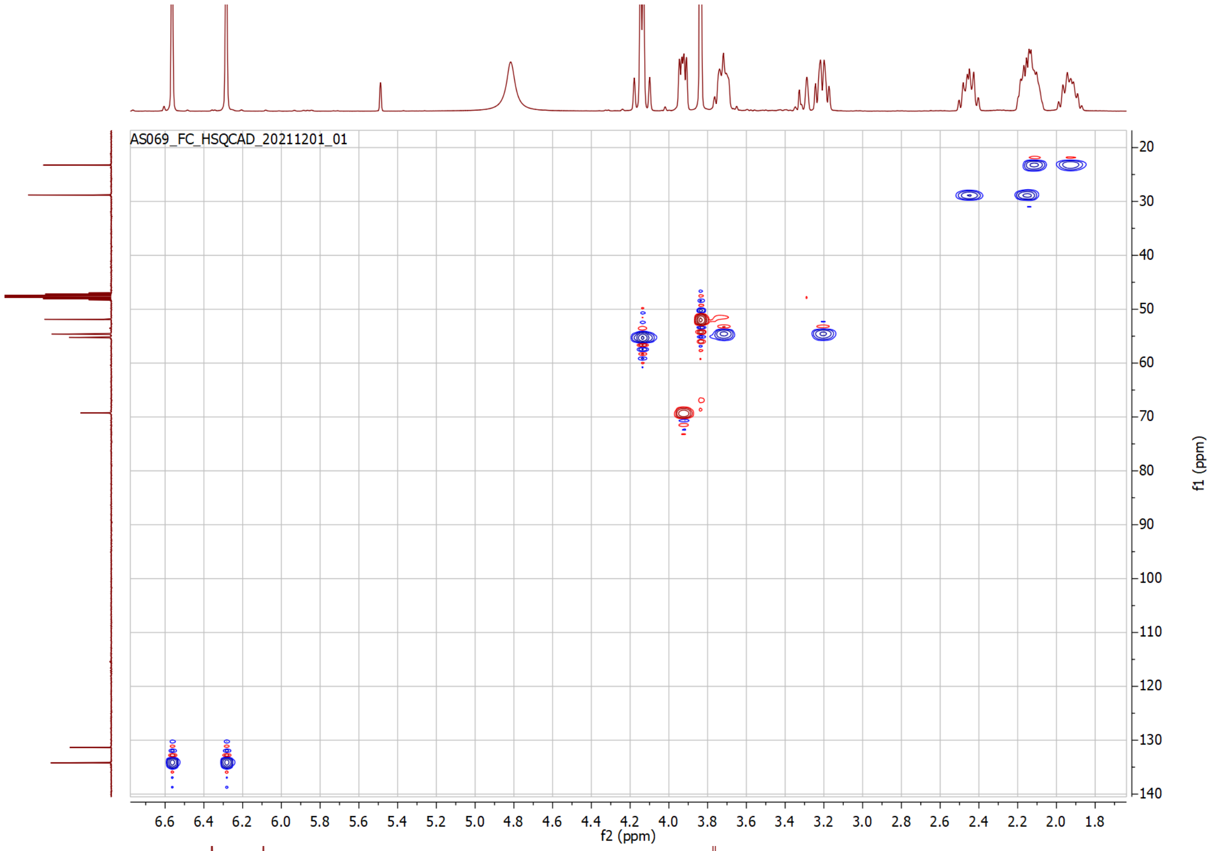
AS069_FC_HSQCAD_20211201_01
f2 (ppm)
f1 (ppm)

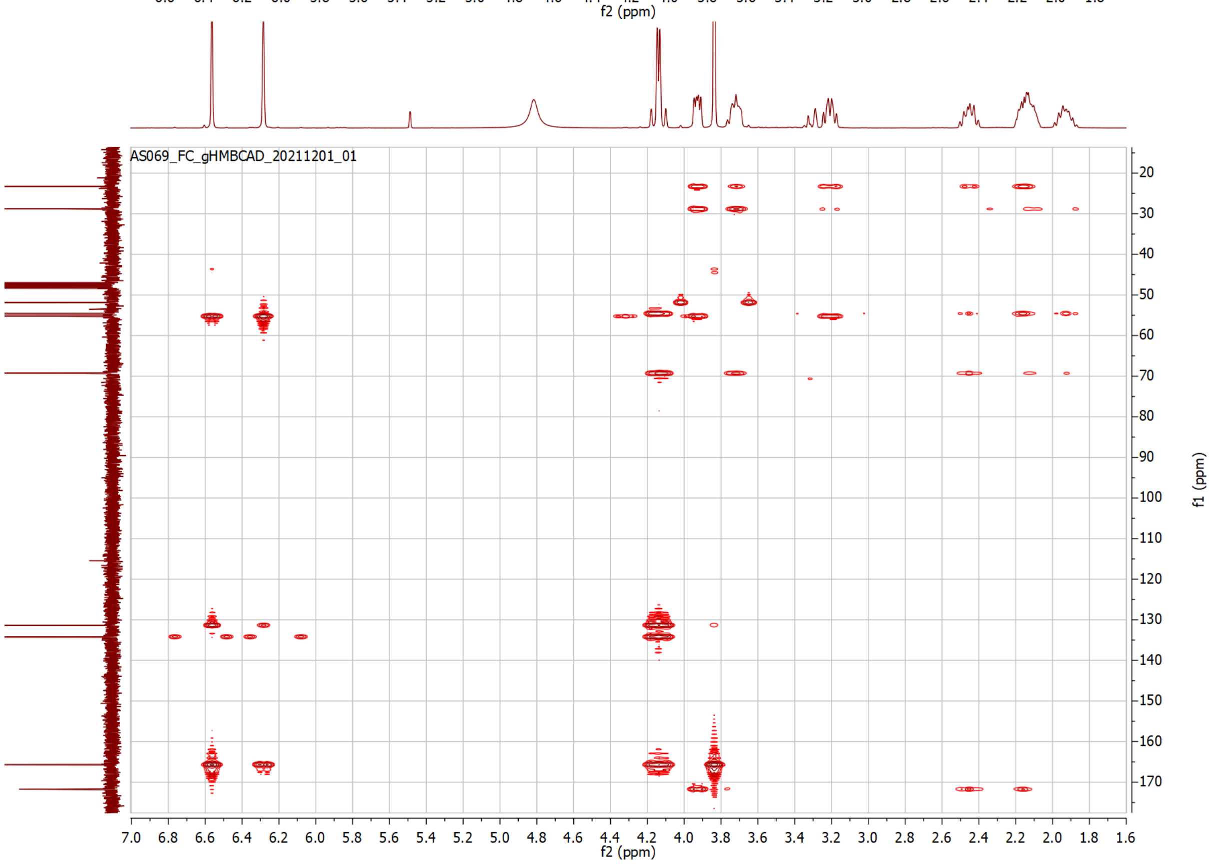
AS069_FC_gHMBCAD_20211201_01
f2 (ppm)
f1 (ppm)

^{1}H-NMR, COSY, ^{13}C and HSQC spectra of 4-((2-(methoxycarbonyl)allyl)thio)benzoic acid (sulfide-MA) in MeOD-d_4:

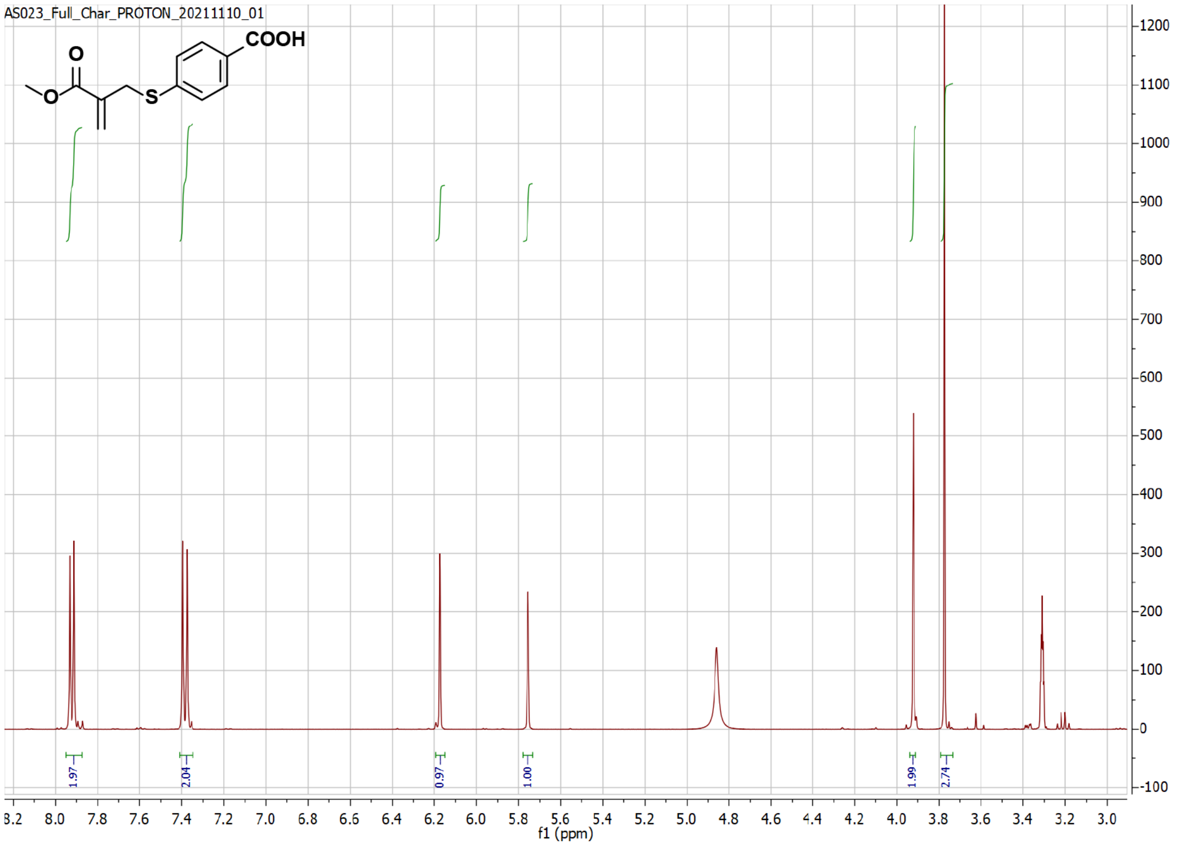

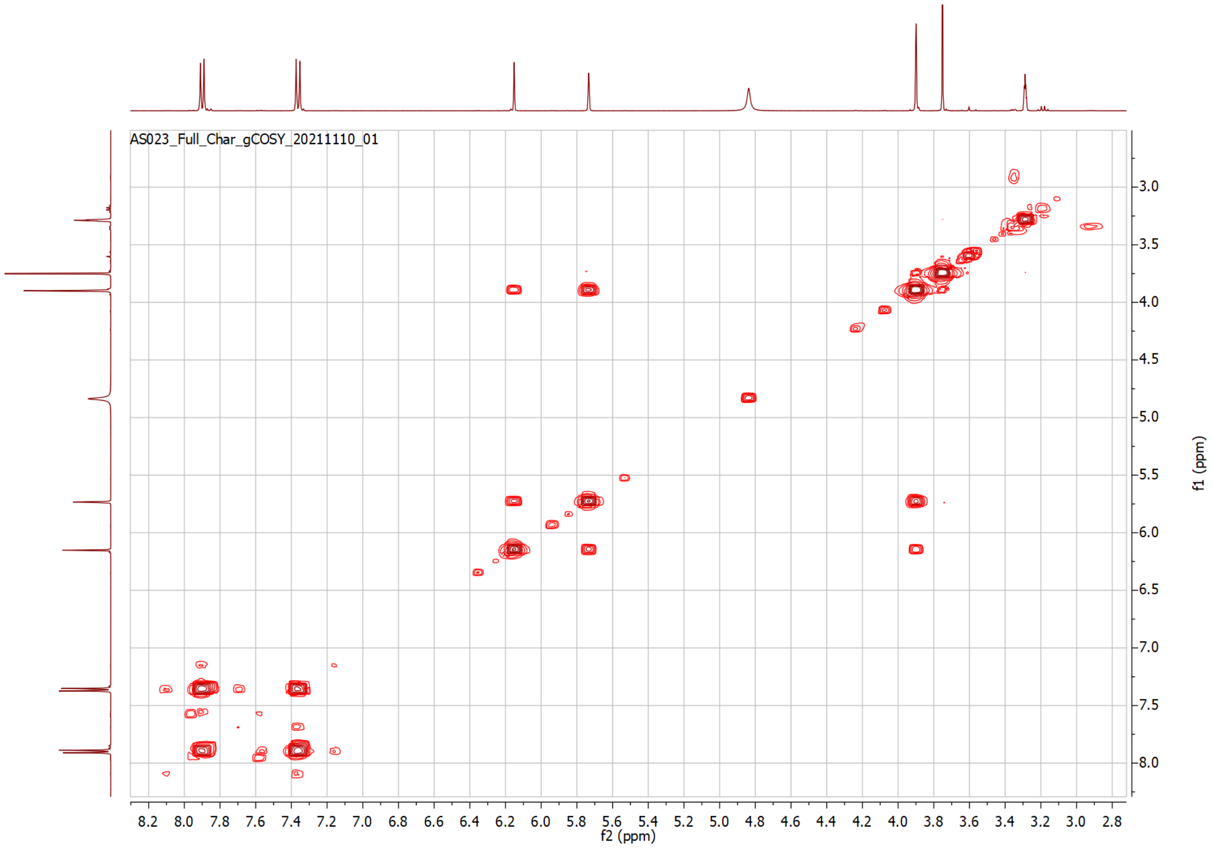

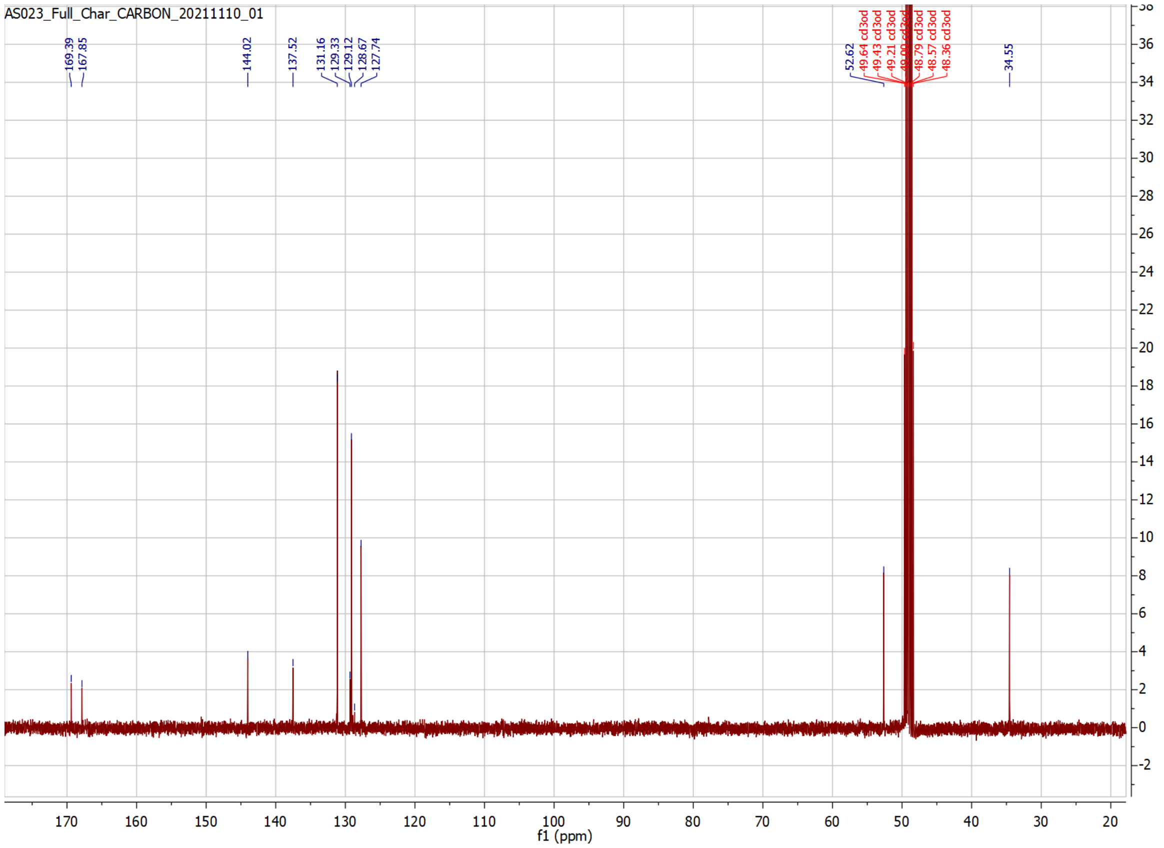
AS023_Full_Char_CARBON_20211110_01
169.39
167.85
144.02
137.52
131.16
129.33
129.12
128.67
127.74
52.62
49.64 cd3od
49.43 cd3od
49.21 cd3od
48.79 cd3od
48.57 cd3od
48.36 cd3od
34.55
f1 (ppm)

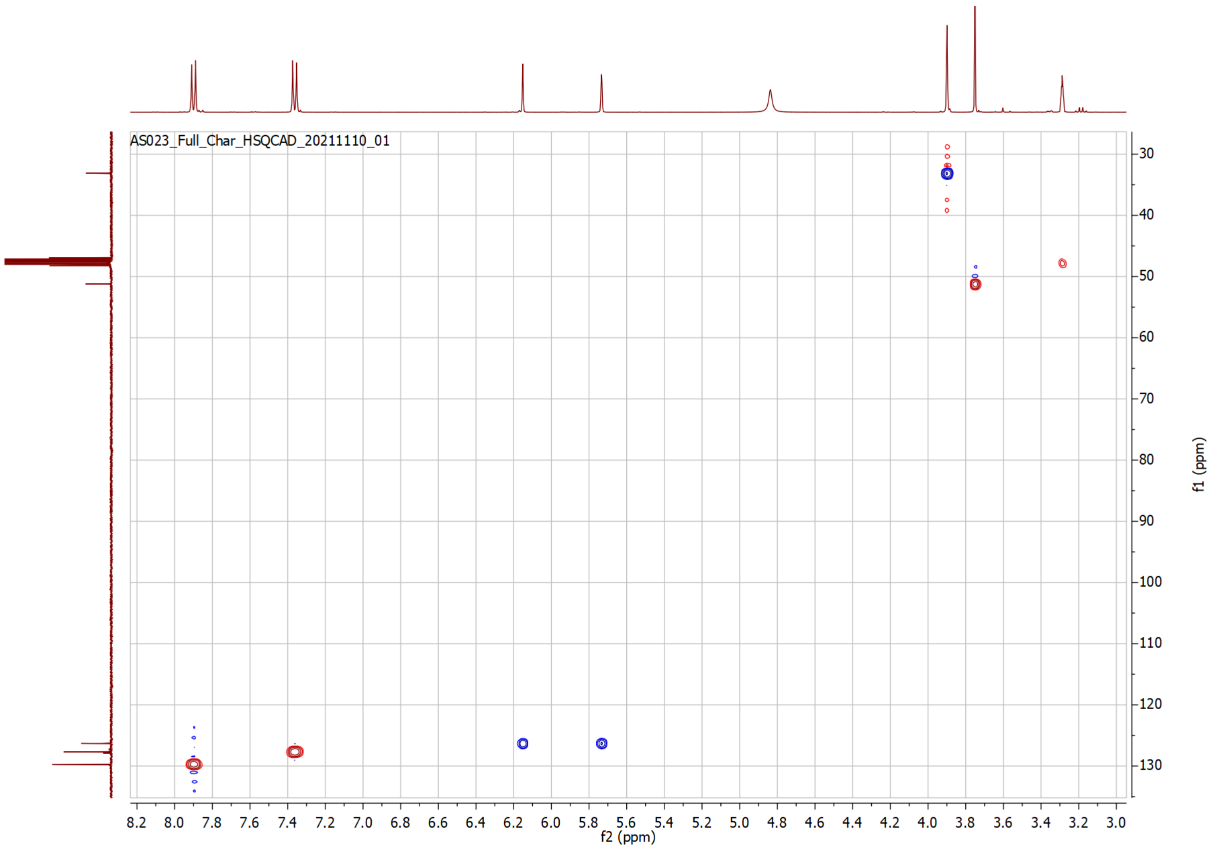
AS023_Full_Char_HSQCAD_20211110_01
f2 (ppm)
f1 (ppm)

^{1}H-NMR, COSY, ^{13}C and HSQC spectra of 4-((2-(methoxycarbonyl)allyl)sulfinyl)benzoic acid (sulfoxide-MA) in MeOD-d_4:

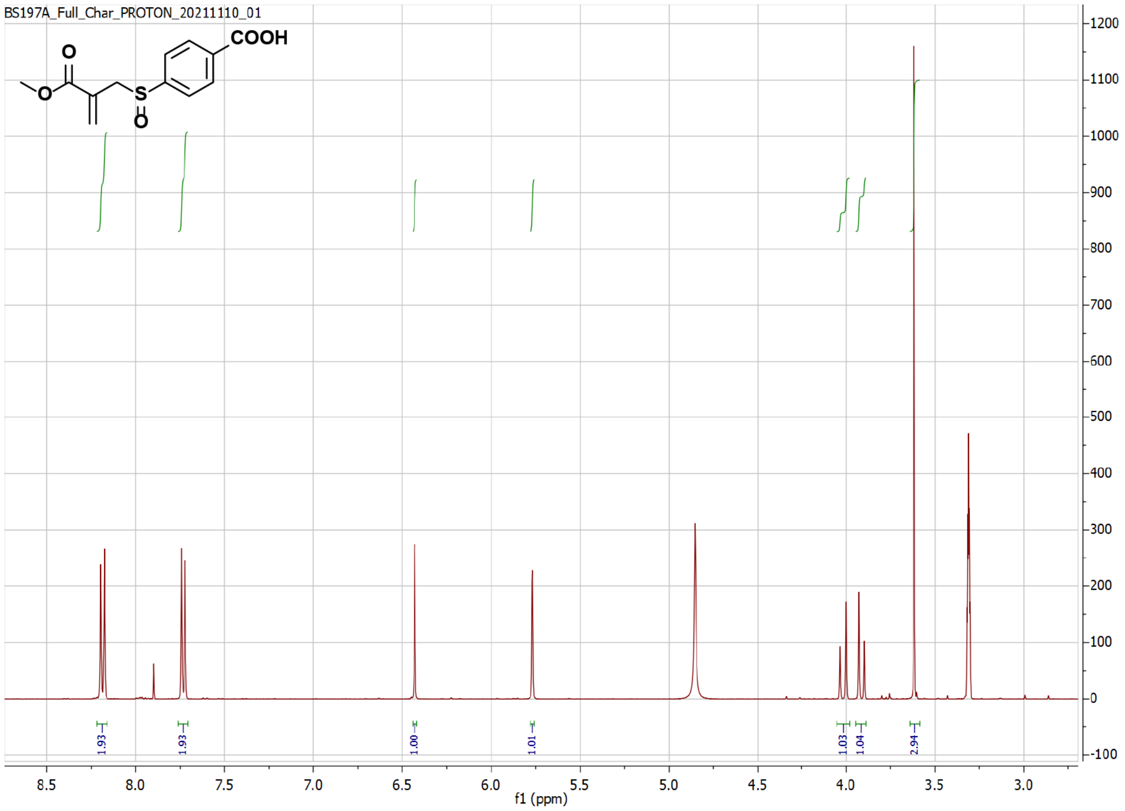

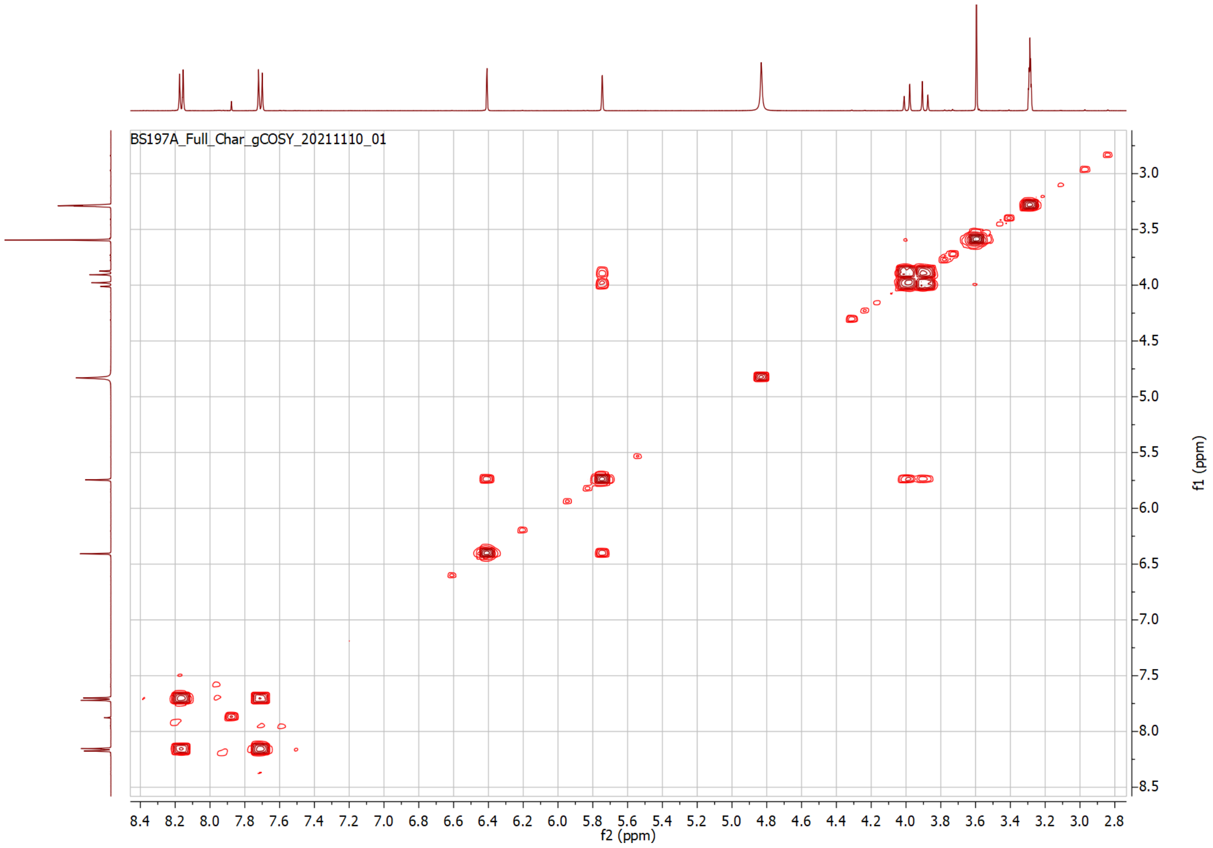

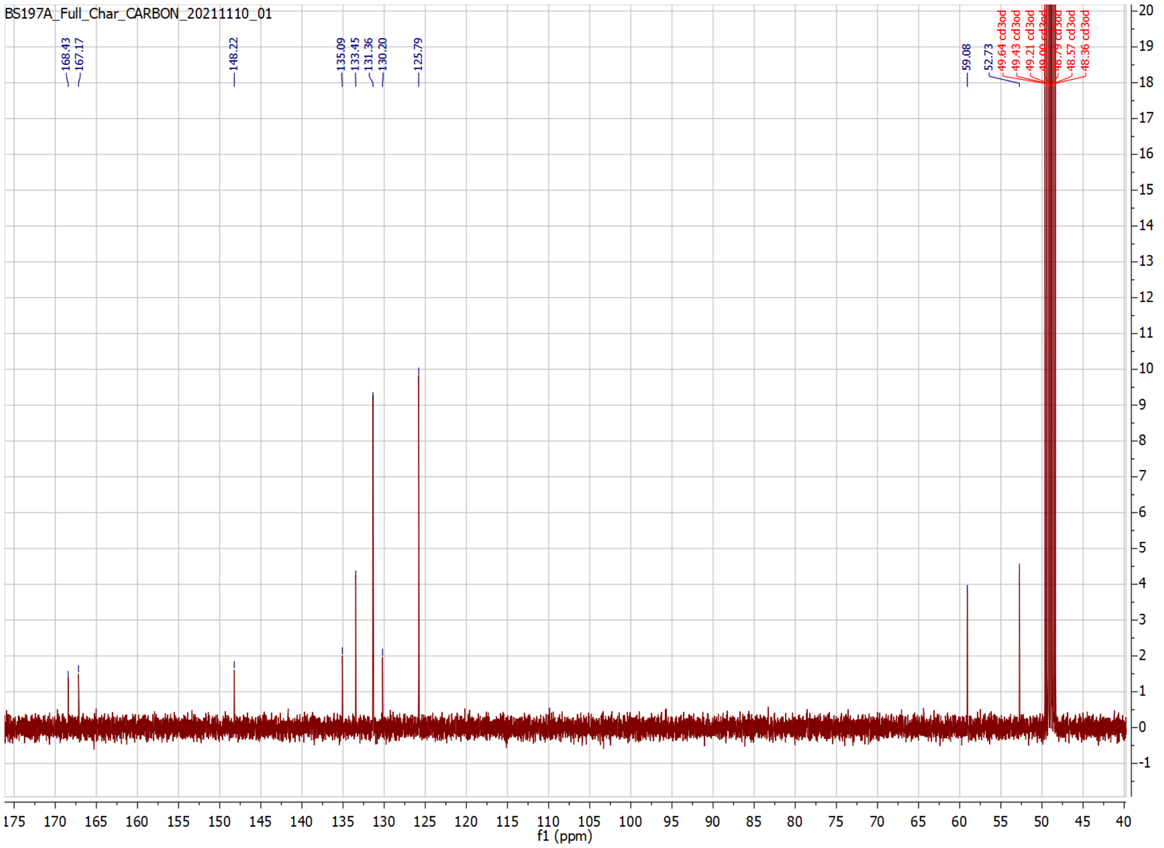
BS197A_Full_Char_CARBON_20211110_01
168.43
167.17
148.22
135.09
133.45
131.36
130.20
125.79
59.08
52.73
49.64 cd3od
49.43 cd3od
49.21 cd3od
49.00 cd3od
48.79 cd3od
48.57 cd3od
48.36 cd3od
f1 (ppm)

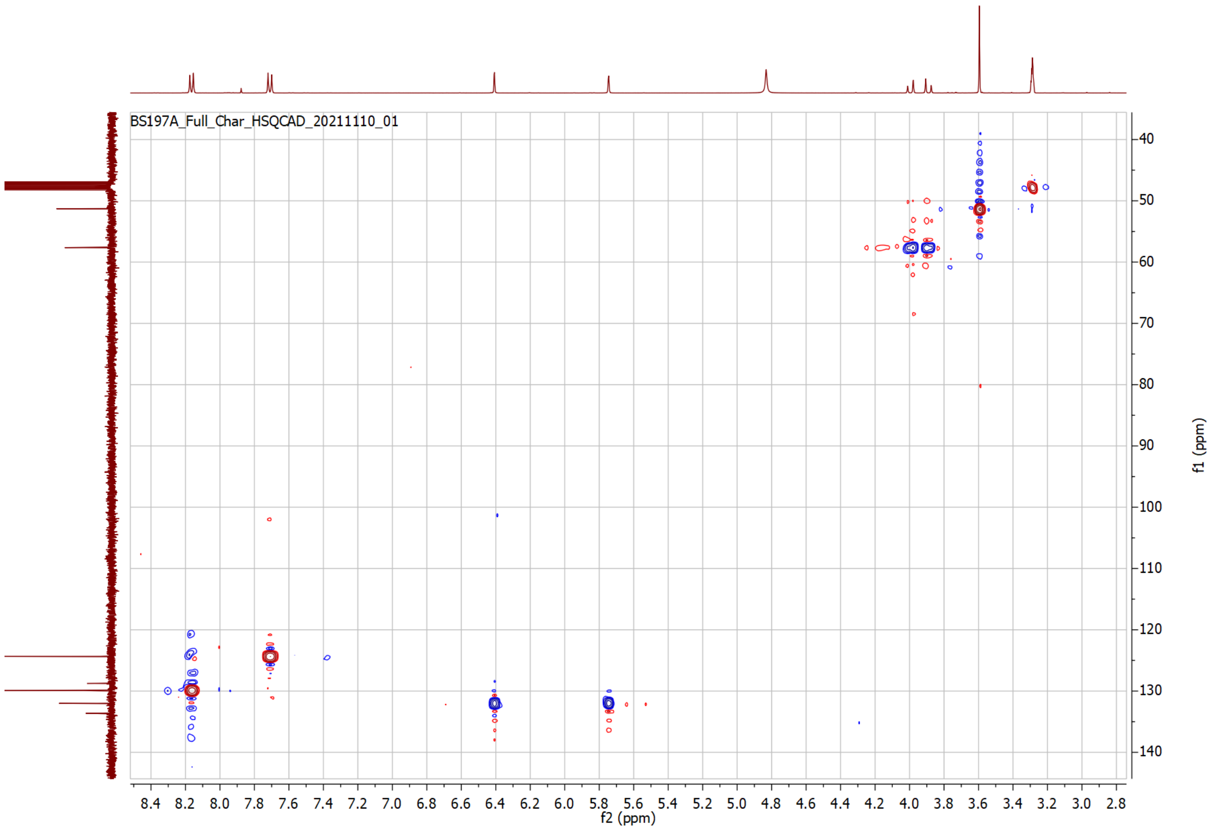
BS197A_Full_Char_HSQCAD_20211110_01
f2 (ppm)
f1 (ppm)

^{1}H-NMR, COSY, ^{13}C, HMBC and HSQC spectra of 4-((2-(methoxycarbonyl)allyl)sulfonyl)benzoic acid (sulfone-MA) in MeOD-d_4:

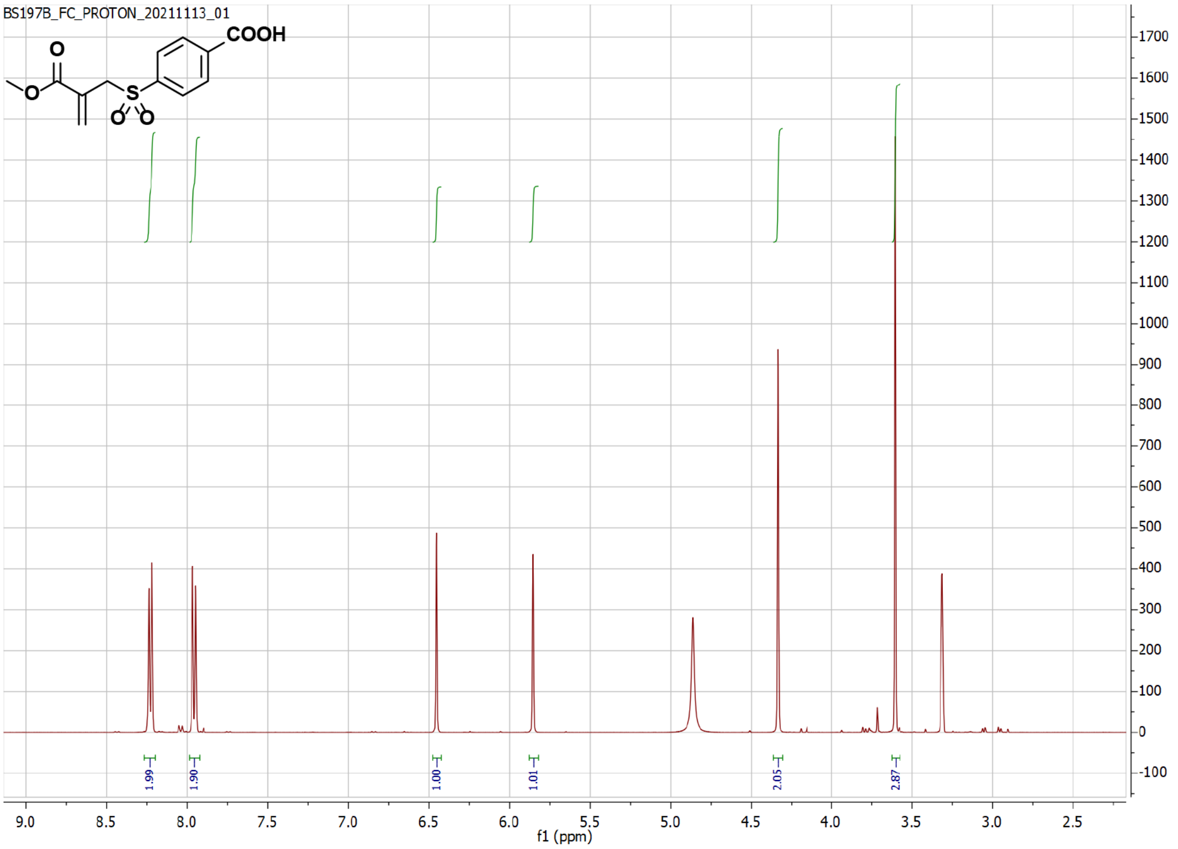

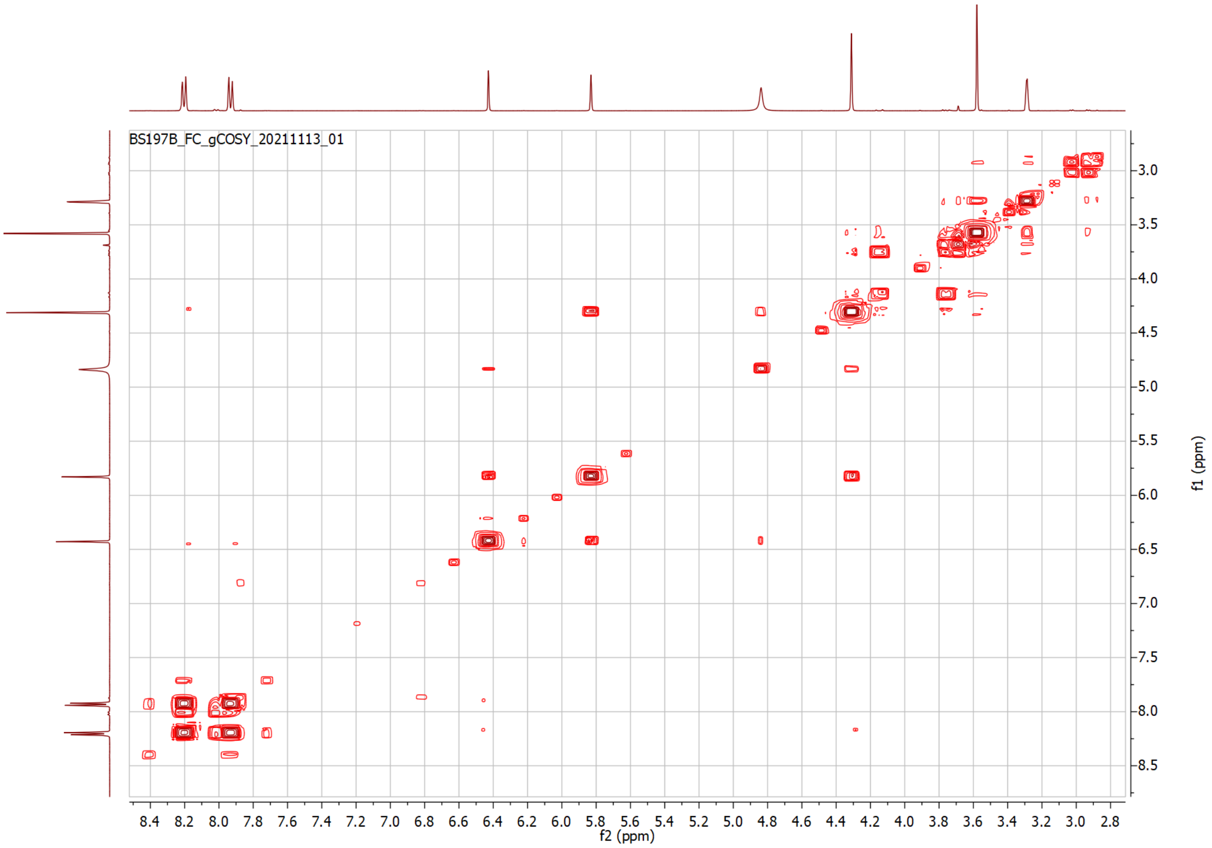

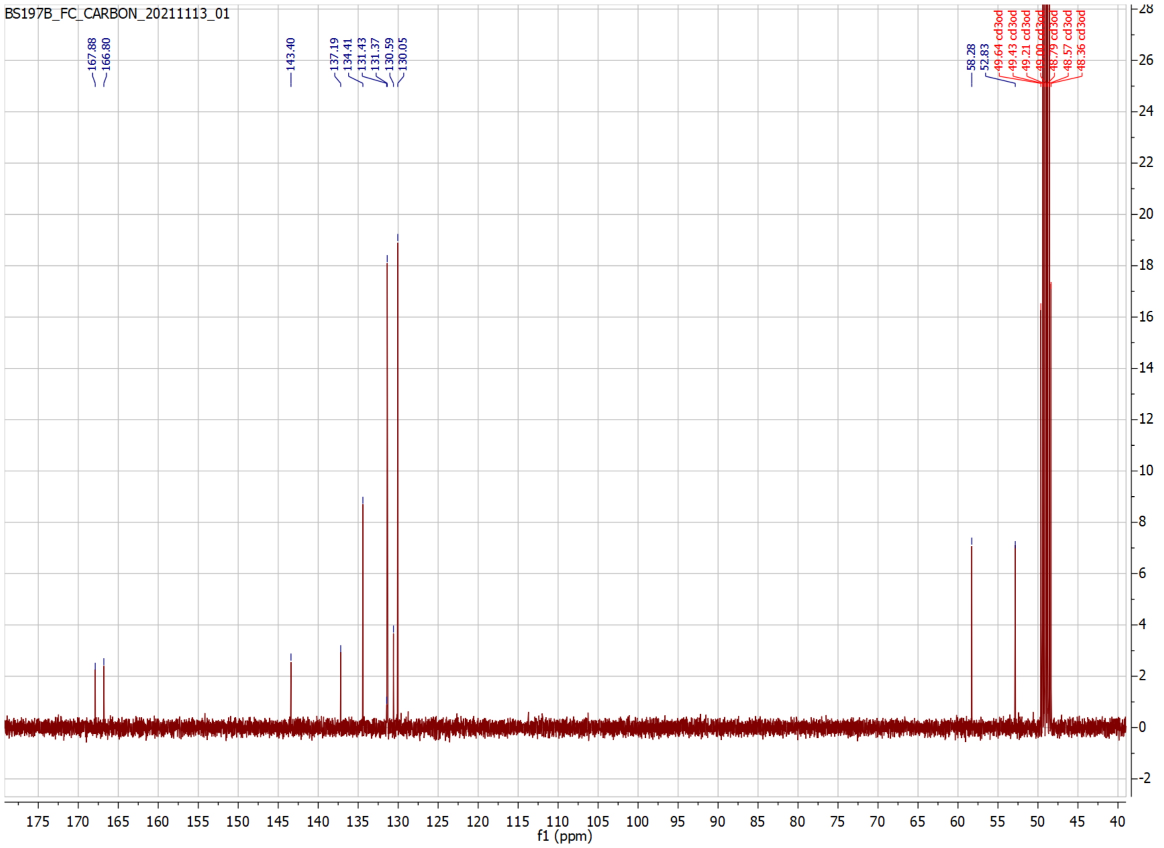
BS197B_FC_CARBON_20211113_01
167.88
166.80
143.40
137.19
134.41
131.43
131.37
130.59
130.05
58.28
52.83
49.64 cd3od
49.43 cd3od
49.21 cd3od
49.00 cd3od
48.79 cd3od
48.57 cd3od
48.36 cd3od
f1 (ppm)

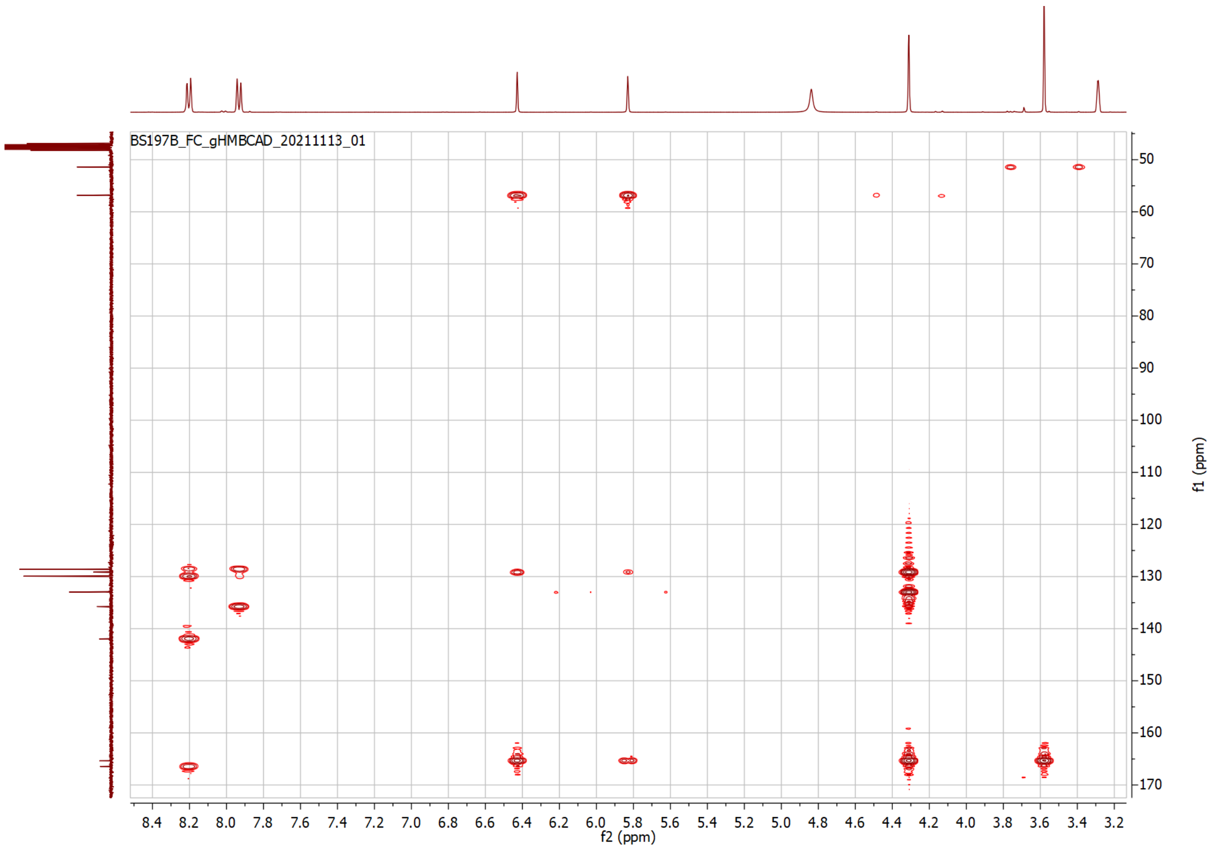
BS197B_FC_gHMBCAD_20211113_01
f2 (ppm)
f1 (ppm)

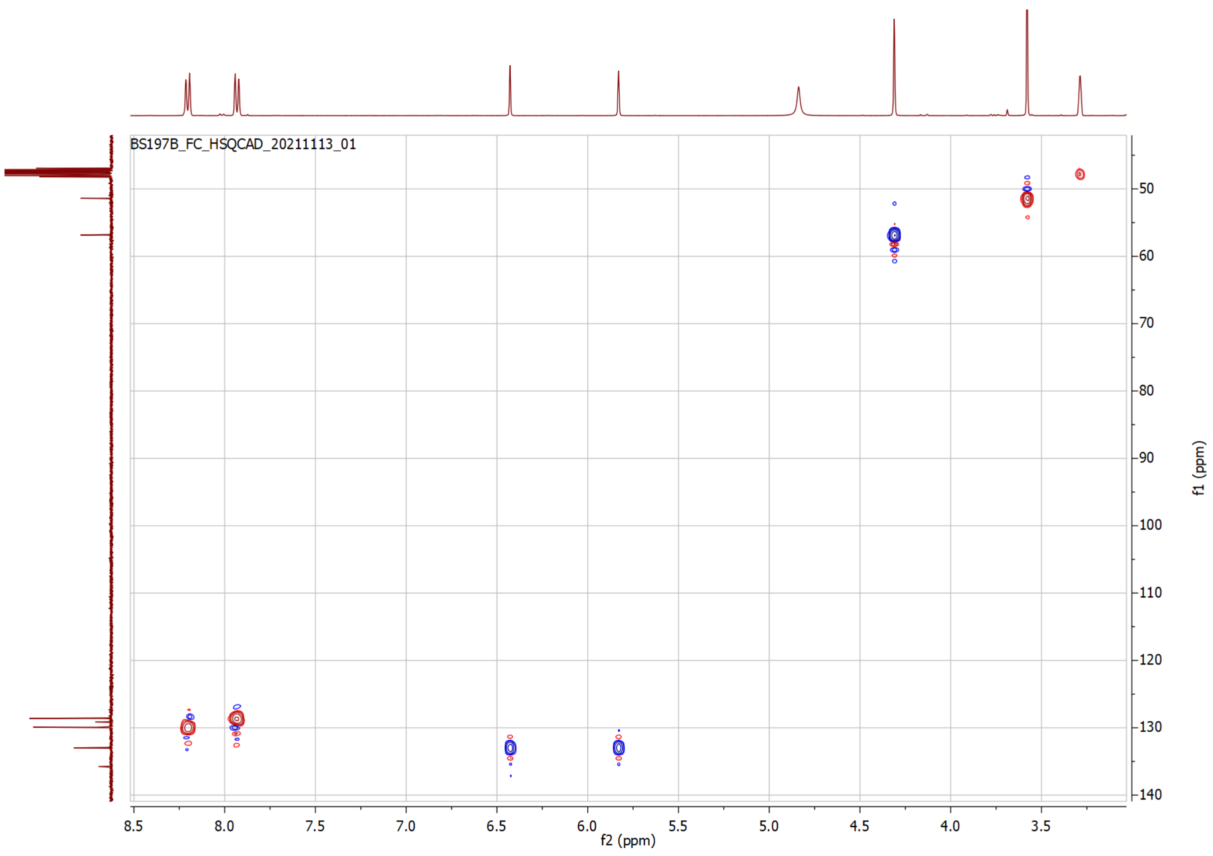
BS197B_FC_HSQCAD_20211113_01
f2 (ppm)
f1 (ppm)
8.5
8.0
7.5
7.0
6.5
6.0
5.5
5.0
4.5
4.0
3.5
50
60
70
80
90
100
110
120
130
140
